# Ferritin-nanocaged aggregation-induced emission nanoaggregates for integrated sensitive detection and treatment of gastric cancer

**DOI:** 10.1016/j.mtbio.2026.102769

**Published:** 2026-01-06

**Authors:** Junjian Deng, Zengxing Zhang, Kejun Li, Yongbin Zheng, Yongfa Zheng

**Affiliations:** a Cancer Center, Renmin Hospital of Wuhan University, Wuhan, 430060, China; b Department of Gastrointestinal Surgery, Renmin Hospital of Wuhan University, Wuhan, 430060, China

**Keywords:** Ferritin, Claudin18.2, AIEgens, Molecular imaging, Gastric cancer

## Abstract

Gastric cancer remains a global health challenge due to late diagnosis and limited targeted therapies. Herein, we report a novel dual-targeted nanoplatform, AIE@HFn-scfv, integrating aggregation-induced emission luminogens (AIEgens) with human heavy-chain ferritin (HFn) conjugated to Claudin18.2-specific single-chain variable fragments (scFv). This nanoconstruct leverages HFn's natural affinity for transferrin receptor 1 (CD71) and scFv-mediated targeting of Claudin18.2 to achieve precise tumor localization. Bioinformatics analysis confirmed co-overexpression of CD71 and Claudin18.2 in gastric cancer tissues, validating their utility as dual targets. Physicochemical characterization revealed stable nanoparticles (∼17 nm) with pH-responsive fluorescence and efficient AIEgens encapsulation. *In vitro* studies demonstrated enhanced cellular uptake in Claudin18.2/CD71-positive MGC803 cells, achieving 52-fold specificity over normal cells. Multimodal imaging in subcutaneous and orthotopic gastric tumor models showed superior tumor-to-background ratios compared to single-target controls, enabling submillimeter tumor detection. Photothermal therapy induced tumor ablation at 53.6 °C, eradicating tumors in 80 % of treated mice without systemic toxicity. Biodistribution studies revealed reduced hepatic accumulation due to dual targeting, enhancing circulation time. This work establishes AIE@HFn-scfv as a promising theranostic platform combining sensitive detection, precise therapy, and biosafety, addressing critical unmet needs in gastric cancer management.

## Graphical abstract

**Figure:**
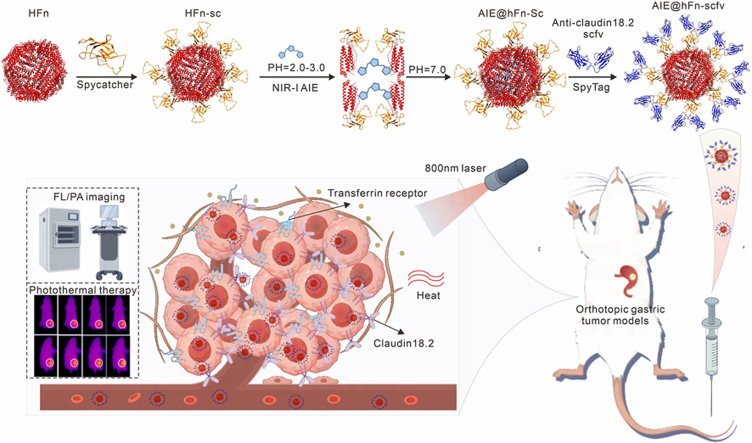

## 1 Introduction

Gastric cancer ranks among the leading causes of cancer-related mortality globally, primarily due to its insidious progression and late-stage diagnosis [1]. Early detection and accurate diagnosis are pivotal for improving patient survival rates, yet current diagnostic modalities face significant limitations in sensitivity, specificity, and the ability to identify tumors at precancerous or early invasive stages [2,3]. Concurrently, the development of effective targeted therapies for gastric cancer remains a formidable challenge, underscoring the urgent need for innovative diagnostic and therapeutic strategies [4,5].

The advent of nanoscale molecular probes and advancements in multimodal optical imaging have revolutionized preclinical cancer diagnostics, with the integration of diagnostic and therapeutic functionalities emerging as a transformative paradigm [6,7]. Molecular probes enable qualitative and quantitative visualization of molecular and cellular processes in real time, offering unprecedented insights into disease pathogenesis [8,9]. Central to the design of effective molecular probes is the identification of cancer-specific biomarkers and the development of contrast agents optimized for multimodal imaging [[10], [11], [12], [13]]. Despite these advances, existing probes for gastric cancer—such as Claudin18.2-targeted PET agents [14], CDH17-targeted nanovesicles [15], and neovascularization-targeting bipyrene-peptide constructs [16]—are hindered by limitations including low sensitivity, high false-positive rates, complex synthesis, and prohibitive costs, impeding their clinical translation. There is a critical need for next-generation nanoprobes characterized by clinical translatability, simplified preparation, and enhanced specificity for early gastric cancer detection and treatment.

To address these challenges, we exploited ferritin as a nanocarrier platform. Human heavy chain ferritin (HFn) has garnered significant attention as a versatile vehicle for cancer theranostics, owing to its inherent biocompatibility, self-assembling hollow nanostructure (12 nm outer diameter, 8 nm inner diameter), and ability to prolong blood circulation by evading renal clearance [[17], [18], [19]]. Furthermore, HFn's natural affinity for transferrin receptor 1 (TfR1/CD71)—a receptor overexpressed in multiple cancer types, including gastric carcinoma—renders it an ideal candidate for targeted delivery [20]. Preclinical studies have demonstrated the efficacy of HFn-based drug carriers in inhibiting tumor growth [19,21], establishing its translational potential.

Complementing the HFn scaffold, we incorporated BBTDT-BT-TPA (aggregation-induced emission luminogens (AIEgens)) as contrast agents [22]. AIEgens exhibit distinct advantages over traditional fluorophores, including robust fluorescence emission in the NIR-II window (900–1700 nm) and photoacoustic tomography capabilities enabled by their photothermal properties [[23], [24], [25]]. These attributes enhance tissue penetration depth and improve signal-to-background ratios while eliminating aggregation-caused quenching (ACQ), a common limitation of conventional organic dyes.

**Scheme 1:**
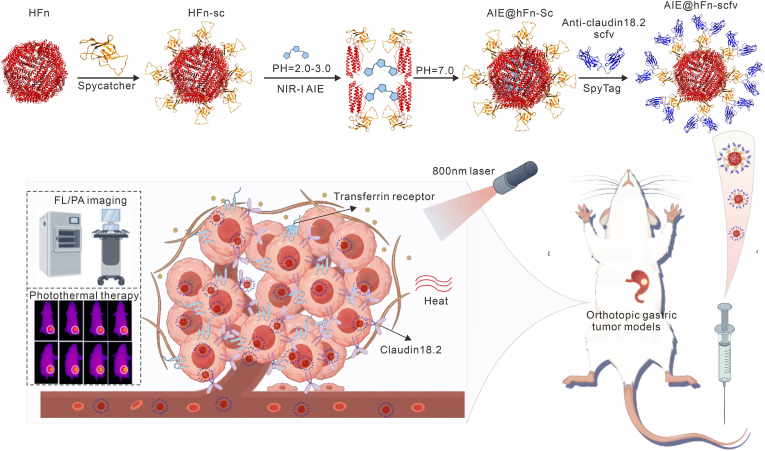
Based on spytag and spycatcher, scfv targeting Claudin18.2 was covalently linked to heavy chain ferritin to form ferritin nanoparticle containing 24 anti-claudin18.2 scfv, which can be used for early targeting of the gastric cancer microenvironment by targeting CD71 with claudin18.2, and the encapsulation of AIE enables multimodal diagnosis of early gastric cancer as well as photothermal therapy.

In this study, we report the design and characterization of a novel multifunctional nanoprobe, AIE@HFn-scfv, which integrates dual targeting specificity for Claudin18.2 and CD71. This nanoplatform leverages HFn's tumor-homing properties and AIEgens' multimodal imaging capabilities to address critical unmet needs in gastric cancer management. By combining receptor-specific single-chain variable fragments (scFv) against Claudin18.2 with HFn's intrinsic CD71 affinity, the probe achieves enhanced tumor specificity and reduced hepatic accumulation. The AIEgens payload enables real-time monitoring of treatment response through fluorescence and photoacoustic (PA) imaging, while the photothermal effect of AIEgens provides a therapeutic modality for targeted tumor ablation(see Scheme 1). Collectively, these features position AIE@HFn-scfv as a promising theranostic tool with the potential to improve sensitive detection, precise therapy, and clinical outcomes in gastric cancer.

## 2 Results

### 2.1 Bioinformatics analysis of CD71 and Claudin18 expression in gastric cancer patients

**Fig. 1:**
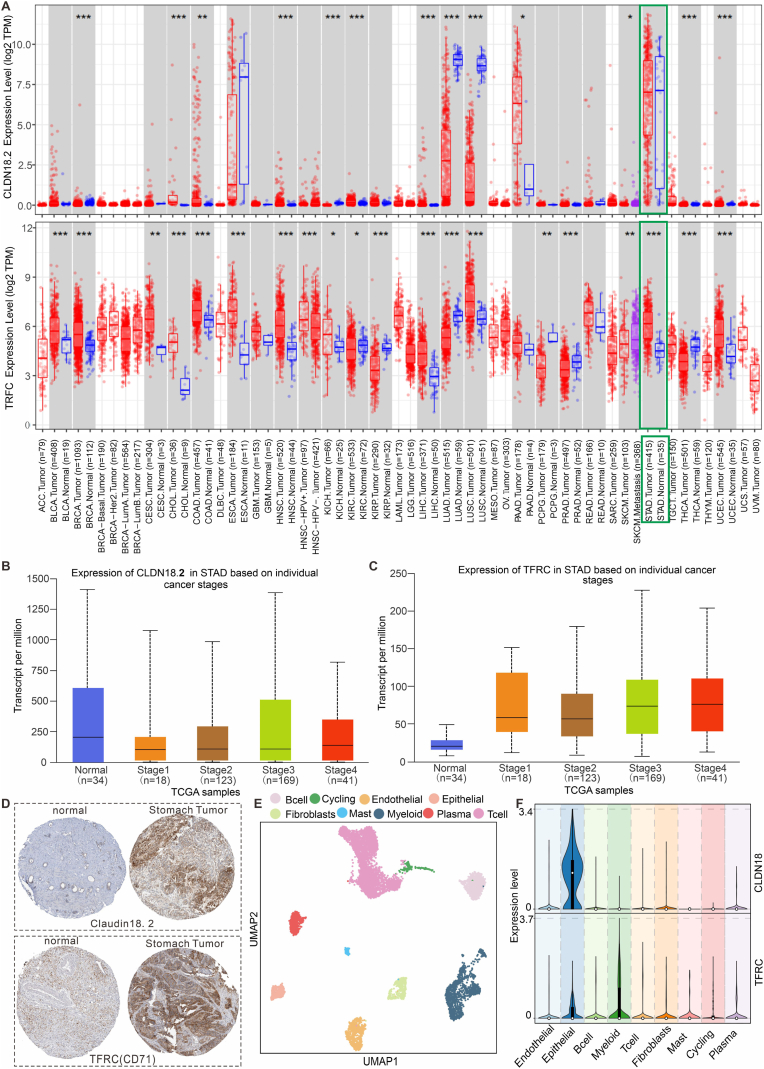
Validation of Claudin18.2 and TFRC(CD71) expression in gastric cancer patients. (A) Analysis of Claudin18 and TFRC expression in different cancer species based on TCGA database. (B–C) Analysis of Claudin18 and TFRC (CD71) molecules expression in gastric cancer patients with different stages based on TCGA database. (D) Analysis of Claudin18 and CD71 expression in tissues of gastric cancer patients based on immunohistochemical results from the Human protein atlas database. (E) Unsupervised clustering algorithm to analyze different cell subpopulations in single-cell data from GSEA gastric cancer patients. (F) Analysis of different cell subpopulations expressing Claudin18 and CD71 in gastric cancer patients.

Claudin18.2 has been used as the target for CAR-T treatment of gastric cancer [7], but early gastric cancer may under-express Claudin18.2 molecule, therefore, choosing another target in combination with molecular probes targeting Claudin18.2 may be more effective for precision diagnosis of early gastric cancer. In this study, we chose the TFRC (CD71) molecule as another gastric cancer target, because the rapid proliferation of tumors cannot be separated from the need for iron element [26,27], and at the same time, the ferritin that interacts with TFRC can self-assemble into nanoparticles of about 10 nm in size, which is the main nanocarrier in the following study. By analyzing the expression of Claudin18.2 and TFRC in the tissue RNA sequence of patients with different clinical cancers, the results of Fig. 1A showed that Claudin18 was mainly expressed in gastric and pancreatic cancers, but the expression was highest in gastric cancers. Meanwhile, CD71 was also highly expressed in gastric cancers, but the expression was lower in pancreatic cancers. Therefore, selecting CD71 and Claudin18.2 as dual targets for gastric cancer may achieve precise targeting of early gastric cancer. Moreover, in order to evaluate whether CD71 and Claudin18.2 are highly expressed in different stages of cancer development, we analyzed the expression of CD71 and Claudin18.2 in gastric cancer patients with different stages, and Fig. 1B–C showed that both molecules were highly expressed in different stages of gastric cancer, demonstrating that CD71 and Claudin18.2 as dual targets are suitable for all stages of gastric cancer development. In order to verify the high expression of CD71 and Claudin18.2 in gastric cancer patients at the protein level, we mined the gastric cancer tissue microarray data in the Human protein atlas database, and confirmed that CD71 and Claudin18.2 were highly expressed in the tumor region, with a significant difference compared to the paraneoplastic expression (Fig. 1D). Besides, we also analyze whether CD71 and Claudin18.2 were expressed only in tumor cells or in other cells of the tumor microenvironment by analyzing a single-cell database of clinical gastric cancer patients (GSE167297), and the cell subpopulations obtained based on an unsupervised clustering algorithm were shown in Fig. 1E. The expression of CD71 and Claudin18.2 in different subpopulations of cells was analyzed, and it was found that these two molecules were mainly expressed in epidermal cells with cancerous properties (Fig. 1F). The above results showed that the dual-targeting property of CD71 and Claudin18.2 as a double-targeting molecular probe is theoretically able to accurately target early gastric cancer tissues.

### 2.2 Physicochemical characterisation of AIE@HFn-scfv

**Fig. 2:**
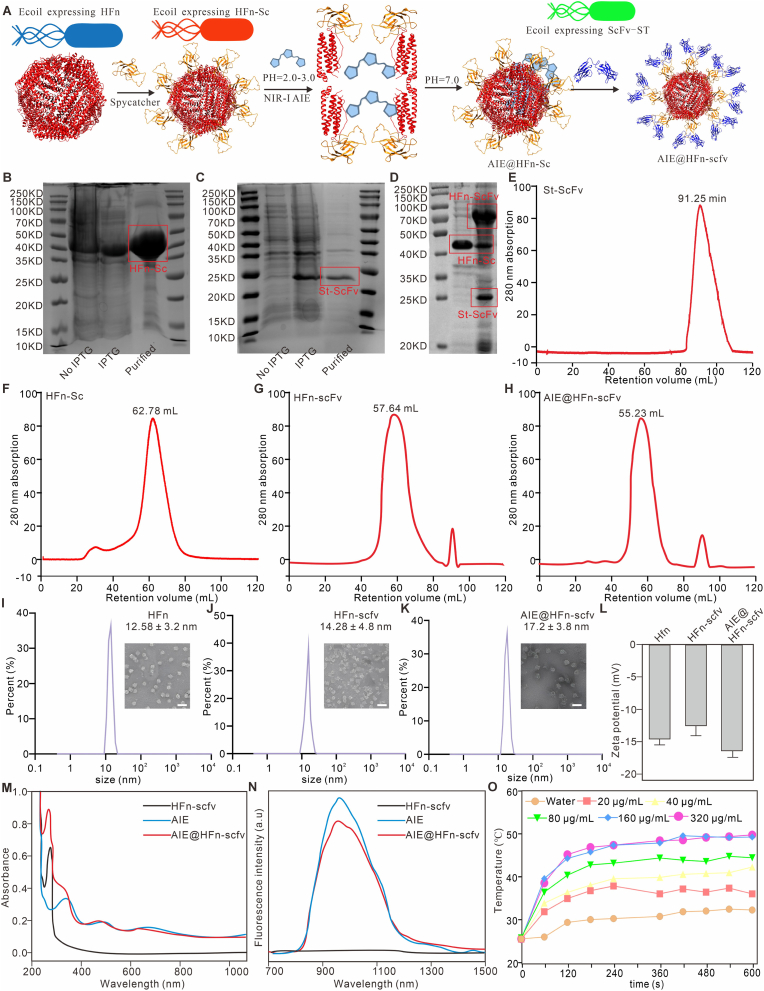
Characterisation of the physico-chemical properties of the AIE@HFn-scfv. (A) Schematic diagram of AIE@HFn-scfv synthesis process. (B) Molecular weight of HFn-sc expressed by prokaryotic bacteria identified by SDS-PAGE. (C) Molecular weight of St-scfv expressed by prokaryotic bacteria identified by SDS-PAGE.(D) SDS-PAGE characterisation of the self-assembly of HFn-scfv and St-scfv in PBS condition. (E–H) Rapid protein liquid chromatography analysis of the purity of HFn-Sc,St-scfv, HFn-scfv and AIE@HFn-scfv. The mobile phase was PBS with a flow rate of 1 mL/min (I–K) Identification of HFn, HFn-scfv and AIE@HFn-scfv sizes using dynamic light scattering and transmission electron microscopy. Scale bar: 20 nm. (L) Zeta potential of HFn, HFn-scfv and AIE@HFn-scfv. (M) UV–Vis curve of HFn, AIE molecular and AIE@HFn-scfv. (N) Fluorescence curve of HFn, AIE molecular and AIE@HFn-scfv (10 μg/mL, based on AIEgen concentration) in PBS (pH = 7.4). Spectra were recorded with an excitation wavelength of 640 nm. (O) Photothermal performance of AIE@HFn-scfv solutions at various concentrations (0.8 W/cm^2^, 808 nm). Data are presented as the mean ± SEM.

In order to realize the dual-targeting property of the nanoprobe for this study against CD71 and Claudin18.2 molecules, we proposed to modify scfv antibodies targeting Claudin18.2 in ferritin nanoparticles, which would achieve 24 scfv molecules loaded in one ferritin nanoparticle, resulting in a multivalent targeting effect. However, due to the unstable tertiary structure of the scfv antibody, the original targeting properties of scfv could not be maintained under the reaction conditions of ferritin-loaded AIEgens, which resulted in the inability to make a fusion protein of anti-Claudin18.2 scfv with ferritin. In order to effectively carry anti-Claudin18.2 scfv, we introduced the Spycatcher003/Spytag003 system into ferritin, which can promote covalent linkage between two different proteins [28,29]. The schedule for the synthesis of AIE@HFn-scfv is shown in Fig. 2A. First, we formed a fusion protein of spycatcher003 with ferritin (HFn-Sc, the amino acid sequence was shown in the Experimental Section), and simultaneously formed a fusion protein of spytag003 with anti-Claudin18.2 scfv (St-scfv), and then the two fusion proteins were obtained by the prokaryotic bacterial expression system. The results in Fig. 2B–C showed that the prokaryotic expression system could express HFn-Sc and St-scfv proteins in large quantities under the induction of Isopropyl-beta-D-thiogalactopyranoside, and the molecular weights of their expression were consistent with the expectation. In order to verify whether the Spycatcher003/Spytag003 system was established in this study, we purified HFn-Sc and St-scfv proteins by affinity chromatography, and co-incubated them at an equimolar concentration in PBS for overnight. The results of SDS-PAGE in Fig. 2D showed that HFn-Sc formed a stable covalently linked protein HFn-scfv with St-scfv.

To evaluate the efficiency of HFn, HFn-scfv, and AIE@HFn-scfv formation, we analyzed these samples with further purification using fast liquid chromatography. The results in Fig. 2E–H show that St-scfv exists as a stable monomer, whereas HFn, HFn-scfv, and AIE@HFn-scfv can self-assemble into nanoparticles for isolation and purification, with HFn-scfv and AIE@HFn-scfv showing peaks earlier than HFn, which proves that the former two are larger than the latter in particle size. We simultaneously analyzed the particle sizes of HFn, HFn-scfv and AIE@HFn-scfv using dynamic light scattering as well as transmission electron microscopy (Fig. 2I–K), and found that the particle size of HFn-scfv, which contains anti-Claudin18.2 scfv, was larger than that of HFn, demonstrating that HFn and anti-Claudin18.2 scfv form a stable covalent bond, and the particle size of AIE@HFn-scfv is larger than that of HFn-scfv, which proves that HFn-scfv can effectively carry AIEgens. Zata potential shows that HFn, HFn-scfv and AIE@HFn-scfv are all negatively charged and do not have significant differences in the charges for each other (Fig. 2L). The chemical structure and ^1^H NMR of BBTDT-BT-TPA (AIEgen) in CDCl_3_ was showed in Figs. S1–S2 (**Supporting information**), and the AIE character of AIEgen was confirmed by a significant fluorescence enhancement in aggregated states (water fraction >70 %) compared to the molecularly dissolved state (Fig. S3, Supporting information). In addition, we also confirmed that the AIEgen has strong photothermal properties (Fig. S4, Supporting information).

To verify that AIEgens was indeed loaded into HFn-scfv nanoparticles, we characterized AIE@HFn-scfv using UV absorption spectra and fluorescence spectra, and the results in Fig. 2M–N show that the UV absorption spectra and fluorescence spectra of AIE@HFn-scfv corresponded to those of AIEgens, and by combining with laser irradiation, we found that the higher concentration of AIE@ HFn-scfv can produce a more enhanced photothermal effect in solution (Fig. 2O). The above results demonstrate that HFn-scfv can effectively load hydrophobic molecules such as AIEgens.

To explore the stability of AIE@HFn-scFv, comprehensive characterizations were performed. Firstly, the size distribution and fluorescence properties of AIE@HFn-scFv under varying pH conditions were analyzed. As shown in Figure S5 A–E (Supporting information), size-exclusion chromatography revealed distinct retention volumes for AIE@HFn-scfv at pH 3, 5, 7, 9, and 11, reflecting pH-dependent structural behaviors. Fig. S5F (Supporting information) further demonstrated pH-responsive fluorescence intensity changes, indicating the material's sensitivity to environmental pH. Stability assessments in aqueous solutions with serum-containing (Fig. S5G, Supporting information) showed that AIE@HFn-scfv maintained structural integrity under different conditions, verified by FITC (HFn-scfv chemically coupled FITC dye) and AIEgens fluorescence signals. Furthermore, long-term stability assessments over 20 days in various biological media confirmed the robustness of AIE@HFn-scfv (Fig. S6, Supporting information). Notably, AIE@HFn-scfv demonstrated exceptional photostability, retaining >90 % of its fluorescence intensity after prolonged laser irradiation, significantly outperforming free AIEgens (Fig. S7, **Supporting information**). In addition, following undergoing five cycles of temperature rise and fall, AIE@HFn-scfv displayed consistent photothermal properties (Fig. S8, **Supporting information**).

Next, the concentration-dependent fluorescence and photoacoustic (PA) properties were evaluated. Fig. S9A–B (Supporting information) illustrated a linear relationship between AIE@HFn-scfv concentration and relative fluorescence intensity or PA signal at 808 nm, confirming reliable quantitative imaging potential. The photothermal effect in Fig. S9C (Supporting information) showed temperature elevation correlated with AIE@HFn-scFv concentration, highlighting its photothermal application potential.

### 2.3 Highly specific targeting and photothermal induction effects of AIE@HFn-scfv on gastric cancer tumor cell lines

**Fig. 3:**
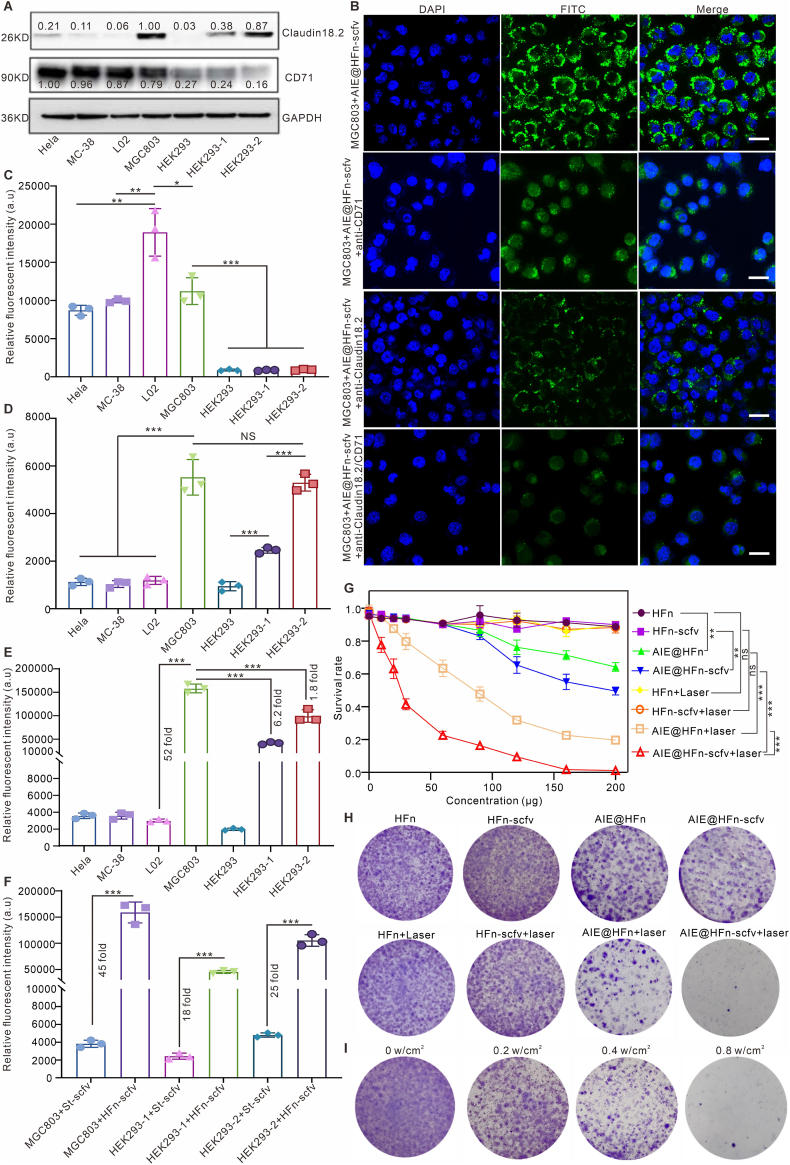
Characterisation of the targeting of AIE@HFn-scfv to different tumor cells in vitro and its killing properties in combination with laser-induced tumor cells apoptosis. (A) Western blotting identifies the expression of CD71 and claudin18.2 in different cells. (B) Confocal imaging identifies that AIE@HFn-scfv-FITC targets gastric cancer cell line MGC803 mainly through CD71 with Claudin18.2 (0.1 mg/mL, based on fusion protein concentration) in culture medium. Scale bar: 20 μm. (C) Flow cytometry statistics of HFn-Sc-FITC uptake by different cells (0.1 mg/mL, based on fusion protein concentration) in culture medium. (D) Flow cytometry statistics of St-scfv-FITC uptake by different cells (0.1 mg/mL, based on fusion protein concentration) in culture medium. (E) Flow cytometry statistics of HFn-scfv-FITC uptake by different cells. (F) Flow cytometry statistics of HFn-scfv-FITC and St-scfv-FITC uptake by MGC803, HEK239-1 and HEK293-2 (0.1 mg/mL, based on fusion protein concentration) in culture medium. (G) CCK8 assay to detect the inhibitory properties of cell proliferation in different indicated treatment groups (0.8 W/cm^2^, 808 nm, 5 min). (H) Crystalline violet staining to assess the characteristics of MGC803 tumor cell killing by different indicated treatment groups (0.1 mg/mL, based on fusion protein concentration) in culture medium (0.8 W/cm^2^, 808 nm, 5 min). (I) Crystalline violet staining identifies a positive correlation between AIE@HFn-scfv and laser power (0.1 mg/mL, based on fusion protein concentration) in culture medium (0.8 W/cm^2^, 808 nm, 5 min). Statistical analysis was performed using one-way ANOVA with Tukey's multiple comparison test. Data are presented as the mean ± SEM. ∗P < 0.05,∗∗P < 0.01, ∗∗∗P < 0.001, and NS: not significant. (For interpretation of the references to color in this figure legend, the reader is referred to the Web version of this article.)

To verify the specific targeting as well as killing effect of AIE@HFn-scfv on gastric cancer tumor cells, we selected tumor cell lines and normal cell lines with different expressions of Claudin18.2 and CD71 molecules (Fig. 3A), including cervical cancer Hela cells, colon cancer MC-38 cells, human-derived normal L02 liver cells, gastric cancer MGC803 cells, human embryonic kidney cells HEK293, HEK293 cells with low expression of Claudin18.2 (HEK293-1), and HEK293 cells with high expression of Claudin18.2 (HEK293-2). To efficiently quantify the uptake of HFn-Sc, St-scfv, and HFn-scfv by the cells, we used FITC isothiocyanate for the labeling of these proteins. We first verified the targeting effect of AIE@HFn-scfv on MGC803. Confocal imaging results (Fig. 3B) showed that AIE@HFn-scfv could be efficiently taken up by MGC803 and mainly stayed in the cytoplasmic region. The targeting effect of AIE@HFn-scfv on MGC803 could be partially blocked by using either anti-claudin18.2 IgG antibody or anti-CD71 IgG antibody to incubate with MGC803 in advance. Meanwhile, it can maximally inhibit the targeting of AIE@HFn-scfv to MGC803 when Claudin18.2 and CD71 molecules are shielded at the same time, which proved that AIE@HFn-scfv could realize the precise targeting of MGC803 by targeting Claudin18.2 and CD71.

To verify the targeting efficacy of HFn protein and anti-Claudin18.2 scfv protein, we co-incubated HFn protein and anti-Claudin18.2 scfv protein with the cells mentioned in Fig. 3A, respectively. The statistics results of flow cytometry in Fig. 3C showed that HFn could efficiently target cell lines with high expression of CD71 and had the highest targeting efficacy to human-derived L02 liver cells, as HFn was reported to primarily target *in vivo* liver cells [30], which corresponded to the results in this study. The statistics results of flow cytometry in Fig. 3D showed that St-scfv mainly targeted cells with high expression of Claudin18.2, and its targeting intensity correlated with the amount of Claudin18.2 expression in tumor cells. Fig. 3E revealed that HFn-scfv could target the gastric cancer MGC803 cell line with high specificity, and its uptake fluorescence intensity was 52 times higher than that of the L02 cell line, and the reason for this phenomenon might be mainly related to the multivalent targeting effect generated by the binding of anti-Claudin18.2 to HFn. To verify the multivalent targeting effect, we used equimolar concentrations of anti-Claudin18.2 scfv and HFn-scfv to co-incubate with HEK293-1 and HEK293-2 cells. As HEK293 lowly expresses CD71, the fluorescence intensity of the uptake of HEK293 is mainly determined by the expression of Claudin18.2. The statistics results of flow cytometry in Fig. 3F showed that the fluorescence intensity of HEK293-1 and HEK293-2 uptake of HFn-scfv was 18-fold and 25-fold of the fluorescence intensity of uptake of anti-Claudin18.2 scfv, respectively. The above results indicated that anti-Claudin18.2 scfv assembled on HFn is more efficient than the fluorescence intensity of anti-Claudin18.2 scfv alone.

To determine that AIEgens loaded on HFn-scfv in combination with laser irradiation can produce a photothermal effect that targets cells for effective cell killing, we used an 808 nm laser to irradiate MGC803 cells incubated with different concentrations of indicated treatments at a power of 0.8 W/cm^2^.The results of CCK8 assay (Fig. 3G) and Crystalline violet staining (Fig. 3H) showed that HFn alone, HFn + Laser, HFn-scfv, and HFn-scfv were unable to kill MGC803 cells, while HFn or HFn-scfv containing AIE has a certain degree of toxic to MGC803, and it was mainly related to the amount of ingested AIEgens. The AIE@HFn and AIE@HFn-scfv groups combined with laser irradiation showed significant killing of MGC803 cells, and the efficiency of cell death induced in the AIE@HFn-scfv + Laser group was much higher than that of AIE@HFn + Laser. The results in Fig. 3I showed that the cell death efficiency of MGC803 cells after incubation with the same concentration of AIE@HFn-scfv mainly showed a positive correlation with the power of laser. The above results confirmed that AIE@HFn-scfv combined with laser irradiation could effectively induce the death of MGC803 cells.

### 2.4 *In vivo* verification of AIE@HFN-scfv targeting subcutaneous gastric cancer model and orthotopic gastric tumor models

**Fig. 4:**
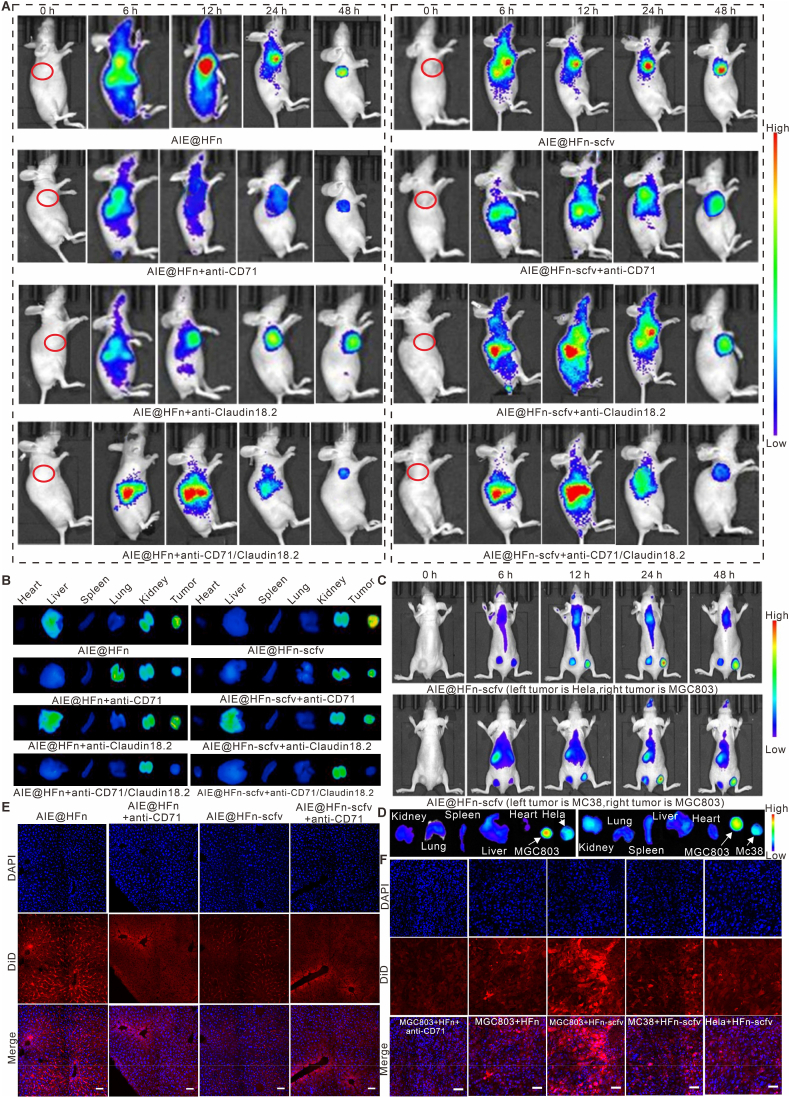
Identification of specific targeting of AIE@HFn-scfv *in vivo* against gastric cancer. (A) IVIS imaging system to observe the targeting effect of different formulated injection groups on MGC803 subcutaneous tumor at different time intervals (Six hours before *i.v.* injection of a total of 0.2 mg of the fusion protein, a total of 0.2 mg of the indicated antibody was injected into the tail vein). (B) IVIS imaging system to observe the distribution of different formulation injection groups in different organs after 48 h. (C) *In vivo* fluorescence imaging showing the specific targeting of intravenously injected AIE@HFn-scfv to MGC803 tumor xenografts over HeLa or MC-38 xenografts in dual-tumor bearing mouse models (*i.v.* injection of a total of 0.2 mg of the fusion protein). (D) IVIS imaging system to observe the distribution of AIE@HFn-scfv in different organs and tumors after 48 h. (E) Immunofluorescence assay to assess the targeting effect of AIE@HFn and AIE@HFn-scfv on liver cells. Scale bar: 100 μm. (F) Immunofluorescence assay to assess the targeting effect of AIE@HFn and AIE@HFn-scfv on different tumor cells. Scale bar: 50 μm.

The specific targeting ability of AIE@HFN-scfv to MGC803 cell line has been verified, but the targeting for *in vivo* tumor model still needs to be determined, because the metabolic characteristics, off-target effects and *in vivo* distribution effect of AIE@HFN-scfv need to be taken into account. We first constructed a subcutaneous tumor model by MGC803 cells, and injected equimolar concentrations of indicated treatment via tail vein 12 days after tumor inoculation, and the whole-body fluorescence intensity of AIEgens in the different groups was acquired by IVIS imaging system. The imaging results of Fig. 4A showed that both AIE@HFn and AIE@HFn-scfv could be highly enriched in the tumor site after 48 h, and the enriched fluorescence intensity of AIE@HFn-scfv in the tumor was significantly higher than that of AIE@HFn. To evaluate whether the tumor target ability of AIE@HFn and AIE@HFn-scfv were dependent on CD71 or Claudin18.2, we injected anti-CD71 IgG antibody as well as anti-Claudin18.2 IgG antibody via intraperitoneal injection 12 h before intravenous injection of AIE@HFn and AIE@HFn-scfv, respectively. Fig. 4A showed that anti-CD71 IgG could shield the targeting properties of AIE@HFn, but would only partially shield the targeting function of AIE@HFn-scfv, whereas anti-Claudin18.2 IgG could not shield the targeting function of AIE@HFn, but would partially shield the targeting function of AIE@HFn-scfv. Simultaneous injection of anti-CD71 IgG and anti-Claudin18.2 IgG significantly shielded the targeting effect of AIE@HFn-scfv in tumors. Dual-receptor blocking experiments confirmed that Claudin18.2 targeting significantly enhances tumor specificity beyond CD71 alone, particularly in gastric cancer models where Claudin18.2 is more selectively overexpressed.

To observe the distribution of AIE@HFn and AIE@HFn-scfv in each organ, we collected organs such as heart, liver, spleen, lung, kidney and tumor from different groups of mice 48 h after intravenous injection of indicated treatment and imaged the fluorescence distribution in different organs using IVIS imaging system. The results in Fig. 4B and Fig. S10A (Supporting information) showed that AIE@HFn and AIE@HFn-scfv could be partially metabolized through the kidney, and AIE@HFn would mostly target the liver region while simultaneously targeting the tumor, but the recruitment effect of AIE@HFn-scfv in the liver was significantly attenuated, which was probably due to the modification of anti-Claudin18.2 scfv shielded part of the targeting effect of HFn itself and enhanced its targeting effect in the tumor region. Pharmacokinetic analysis revealed a circulation half-life of ∼8.9 h for AIE@HFn-scfv, significantly longer than that of free AIEgens (∼1.4 h) or AIE@HFn (∼6.5 h), corroborating its enhanced tumor accumulation and reduced off-target effects (Fig. S11, Supporting information). Tissue immunofluorescence results (Fig. 4E–F) against liver and tumor of different groups of mice also confirmed that AIE@HFn could target liver cells, whereas AIE@HFn-scfv had a weaker targeting effect in the liver region and a stronger targeting effect in MGC803 tumor. These results showed that AIE@HFn-scfv could be efficiently enriched in the tumor area through the targeting effect of CD71 and Claudin18.2, and could effectively circumvent recruitment in the liver region and enhance circulation time in the peripheral blood.

In order to verify the specific targeting of AIE@HFn-scfv to gastric cancer, we also constructed a tumor-bearing mouse model with different types of tumors, and evaluated its specific targeting by observing the recruitment effect of AIE@HFn-scfv in different types of tumors. The results of *in vivo* and in vitro organ fluorescence images in Fig. 4C–D and Fig. S10B (Supporting information) showed that AIE@HFn-scfv could efficiently target the tumor model constructed by MGC803 cell line, while the tumor model constructed by Hela cell line and MC-38 cell line was weak. In addition, the immunofluorescence images of different types of tumors in Fig. 4F also showed that AIE@HFn-scfv mainly targeted MGC803 cell lines.

**Fig. 5:**
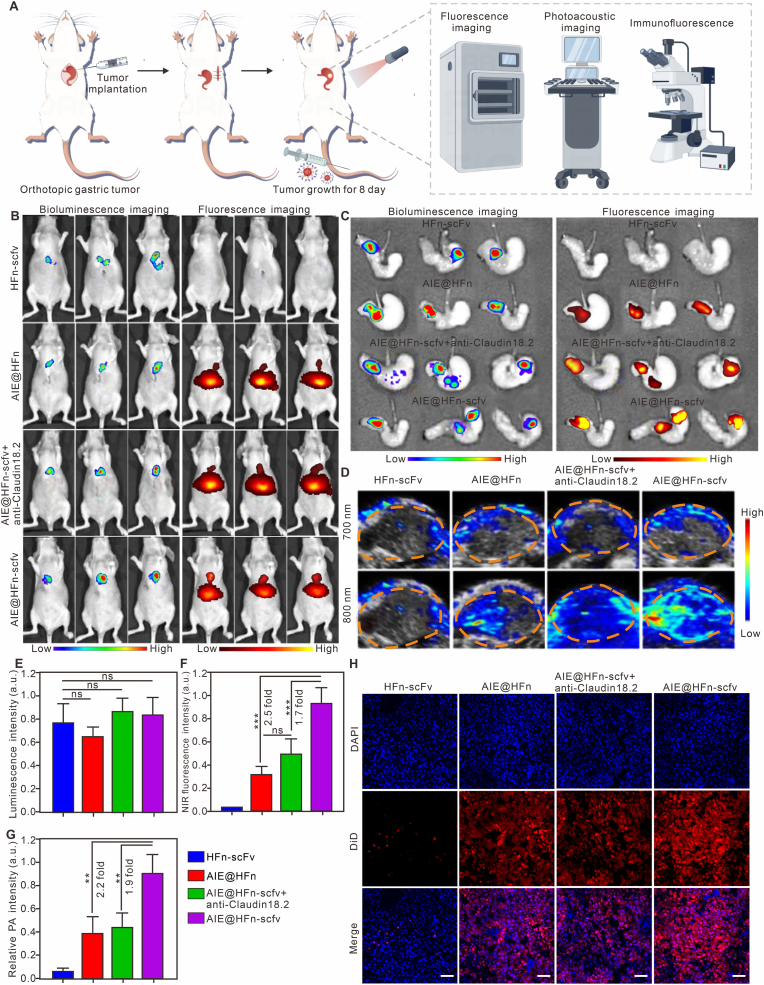
Comprehensive imaging evaluation of AIE@HFn-scfv in orthotopic gastric tumor models. (A) Schematic illustration of the experimental procedure: orthotopic gastric tumor implantation, and application of fluorescence imaging, photoacoustic imaging, and immunofluorescence techniques. (B) *In vivo* bioluminescence and fluorescence imaging of tumor-bearing mice treated with HFn-scfv, AIE@HFn, AIE@HFn-scfv + anti-Claudin 18.2 antibody, and AIE@HFn-scFv (Six hours before *i.v.* injection of a total of 0.2 mg of the fusion protein, a total of 0.2 mg of the indicated antibody was injected into the tail vein). (C) *Ex vivo* bioluminescence and fluorescence imaging of stomach harvested from mice in different groups. (D) Photoacoustic imaging of tumors in mice at 700 nm and 808 nm wavelengths. (E–G) Quantitative analysis of luminescence intensity (E), NIR fluorescence intensity (F), and relative PA intensity (G) in different groups. (H) Immunofluorescence staining of tumor tissues: blue (DAPI, nucleus), red (DiO, imaging agents), and merged images. Scale bar: 50 μm. Statistical analysis was performed using one-way ANOVA with Tukey's multiple comparison test. Data are presented as the mean ± SEM. ∗P < 0.05,∗∗P < 0.01, ∗∗∗P < 0.001, and NS: not significant. (For interpretation of the references to color in this figure legend, the reader is referred to the Web version of this article.)

To systematically assess the imaging performance of AIE@HFn-scfv in orthotopic gastric tumor models, a series of imaging analyses were conducted. As depicted in Fig. 5A, the experimental workflow involved orthotopic gastric tumor implantation, and subsequent application of fluorescence imaging, photoacoustic imaging, and immunofluorescence techniques. Firstly, *in vivo* bioluminescence and fluorescence imaging (Fig. 5B) revealed distinct signals in tumor-bearing mice: mice treated with AIE@HFn-scfv exhibited stronger tumor-region fluorescence signals compared to other groups (HFn-scfv, AIE@HFn, AIE@HFn-scfv + anti-Claudin 18.2), indicating the conjugates’ specific targeting ability. This specificity was further validated by *ex vivo* bioluminescence and fluorescence imaging of excised stomach from different group (Fig. 5C), where tumor tissues from the AIE@HFn-scfv group showed remarkably higher signal intensity. Subsequently, photoacoustic (PA) imaging results (Fig. 5D) demonstrated enhanced tumor visualization in the AIE@HFn-scfv group at 808 nm wavelengths, reflecting superior tumor penetration and contrast. *Although the absorption at* 808nm *appears as a tail in the spectrum, it is effectively leveraged for generating strong photoacoustic signals due to the high sensitivity of PA imaging and efficient tumor accumulation.* Furthermore, the statistical analyses (Fig. 5E–G) quantitatively supported these findings: the AIE@HFn-scfv group exhibited a 2.5-fold increase in NIR fluorescence intensity and a 2.2-fold increase in PA intensity versus AIE@HFn groups. Additionally, immunofluorescence staining (Fig. 5H) confirmed cellular uptake and targeting efficiency, as the AIE@HFn-scfv group displayed stronger red fluorescence (DiD) in tumor cells, evidencing effective agent accumulation in the tumor microenvironment. Collectively, these integrated imaging and quantitative analyses highlight the superior targeting and imaging capabilities of AIE@HFn-scfv in orthotopic gastric tumor models.

### 2.5 Advantages of AIE@HFN-scfv *in vivo* multimodal imaging and photothermal induction effect

**Fig. 6:**
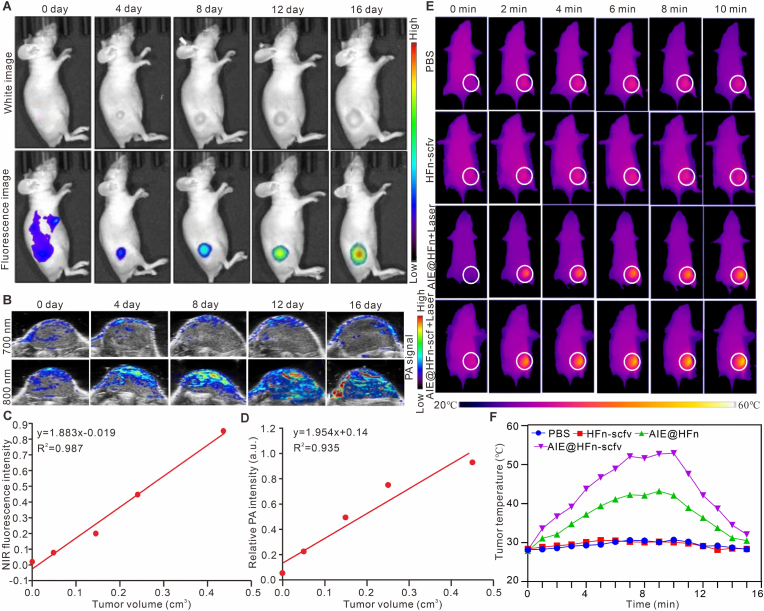
AIE@HFn-scfv combines fluorescence imaging and photoacoustic imaging to enable diagnosis of early, mid and late gastric cancer tumors and *in vivo* induction of photothermal phenomena. (A) IVIS imaging system to assessment of the diagnostic effect of AIE@HFn-scfv in nude mice loaded with MGC803 tumor of different sizes at 24 h post-injection. (B) PA imaging system to assessment of the diagnostic effect of AIE@HFn-scfv in nude mice loaded with MGC803 tumor of different sizes at 24 h post-injection. (C) Assessment of linear correlation between tumor size and detected fluorescence intensity. (D) Assessment of linear correlation between tumor size and detected PA signal intensity. (E) visual FLIR system was used to evaluate the intensity of the photothermal effect produced by combining lasers in different designated treatment groups (0.8 W/cm^2^, 808 nm). (F) Statistics of temperature change curves for different indicated treatment groups. A total of 0.2 mg of the indicated fusion protein was injected into the tail vein (0.8 W/cm^2^, 808 nm).

In order to verify the multimode imaging characteristics of AIE@HFn-scfv and its diagnostic effect on early gastric cancer, we performed fluorescence imaging and photoacoustic imaging on tumor-bearing mice with different tumor sizes. The results of fluorescence imaging (Fig. 6A and Fig. S12A (Supporting information)) showed that the fluorescence intensity enriched in the tumor increased significantly with the increase of the tumor size, and the earliest time to detect the tumor fluorescence intensity was the fourth day after inoculation of the tumor. The linear correlation analysis of Fig. 6C shows that there is a positive correlation between the tumor volume and the fluorescence intensity in the tumor, and the R square value is 0.987. In addition, in order to obtain the photoacoustic signal intensity in tumors of different sizes, we first use the 700 nm laser which does not excite the photothermal effect of AIEgens to deduct the photoacoustic background signal intensity of the tumor itself, and then use the 808 nm laser to effectively excite the photoacoustic signal intensity of AIEgens. The photoacoustic imaging results of Fig. 6B and Fig. S12B (Supporting information) show that the PA signal excited by 808 nm laser is much stronger than that excited by 700 nm laser, and the PA signal increases gradually with the increase of the tumor. Meanwhile, the linear correlation analysis of Fig. 6D also showed that there was a positive correlation between tumor size and PA signal intensity in the tumor, and its R square value was 0.935. The above results prove that AIE@HFn-scfv has good fluorescence imaging and photoacoustic imaging effect.

### 2.6 Efficacy and biosafety of AIE@HFN-scfv *in vivo* photothermal therapy

**Fig. 7:**
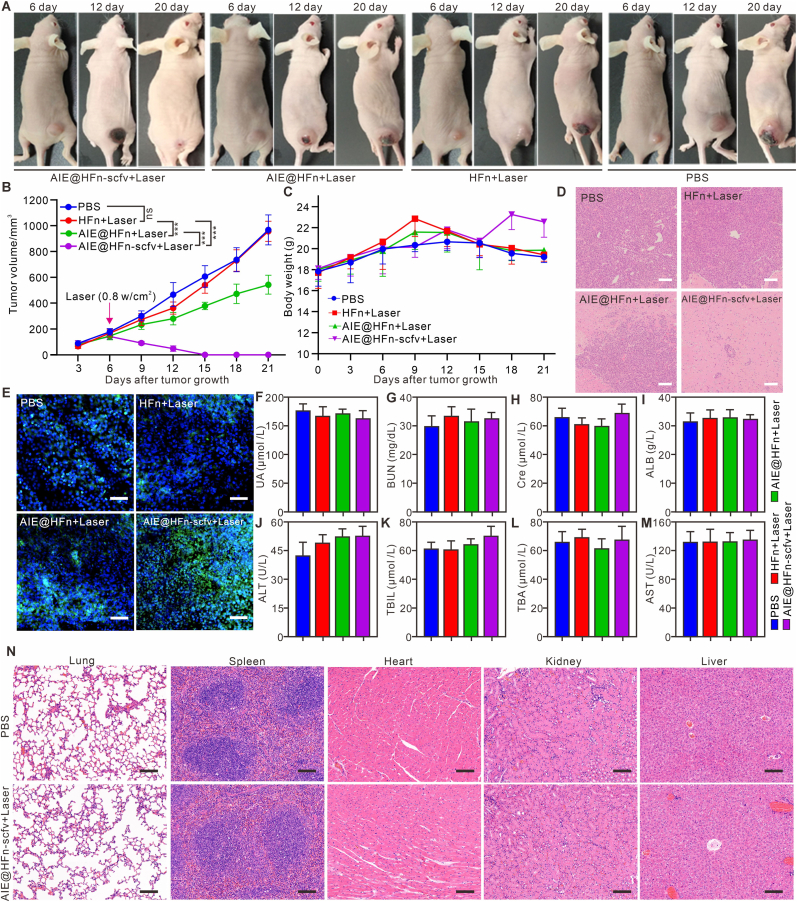
AIE@HFn-scfv combined with laser irradiation enables early treatment of *in vivo* induced gastric cancer tumors and biosafety assessment. (A) Assessment of tumor therapeutic efficacy in nude mice treated with subcutaneous inoculation of MGC803 tumors by different indicated treatment groups (A total of 0.2 mg of the indicated fusion protein was injected into the tail vein, (0.8 W/cm^2^, 808 nm, 10 min)). (B) Statistics of tumor growth curves of MGC803 tumor in different indicated treatment groups, and the laser irradiation was performed on day 8 after tumor inoculation, the irradiation condition was 0.8W/cm^2^, and the irradiation time was 10 min. (C) Statistics of body weight in different indicated treatment groups. (D) Hematoxylin−eosin (H&E) staining of tumor sections of different treatment groups after 14 days. Scale bar: 100 μm. (E) Apoptosis tunnel staining staining of tumor sections of different treatment groups after 14 days. (F-M)Statistics of liver and kidney function indicators (UA (F), BUN (G), CRE (H), ALB (I), ALT (J), TBIL (K), TBA (L) and AST (M) of peripheral blood from mice with different indicated treatment. (N) H&Estained tissues of heart, liver, spleen, lung, and kidney of mice from the PBS and AIE@HFn-scfv + laser group. Scale bar: 100 μm. Data are presented as the mean ± SEM. UA: urea, BUN:blood urea nitrogen, Cre: Creatinine, ALB: albumin, ALT: alanine aminotransferase, TBIL:total bilirubin, ALP: alkaline phosphatase; TBA: total bile acid, AST: aspartate aminotransferase.

In order to verify the photothermal effect induced by AIE@HFn-scfv in the tumor area, we irradiated the tumor area of tumor-bearing mice with 808 nm laser at a power of 0.8 W/cm^2^ for 10 min. The temperature of the tumor area was recorded by infrared thermal imager every other minute. The related results are shown in Fig. 6E–F. The average maximum temperature of tumor area in PBS group and mice injected intravenously with HFn was 31.2 °C, while that in mice injected intravenously with AIE@HFn was 41.3 °C, and that in mice injected intravenously with AIE@HFn-scfv was 53.6 °C. The above results confirm that AIE@HFn-scfv can effectively promote the recruitment of AIEgens in tumor area and induce efficient photothermal effect. To verify the therapeutic effect of AIE@HFn-scfv combined with laser irradiation on MGC803 tumor-bearing mice, we injected indicated treatment intravenously on the 5th day, and irradiated the mice on the 6th day. The tumor treatment Physicalv image of Fig. 7A and the tumor growth curve of Fig. 7B show that AIE@HFn-scfv can effectively heal tumor-bearing mice, and the treatment has no effect on the body weight of tumor-bearing mice (Fig. 7C). In order to verify the residual tumor cells in the tumor area, we get the tumor area sample on the 20th day of tumor inoculation and processed with H&E staining. The results of Fig. 7D show that there were almost no tumor cells in the AIE@HFn-scfv + Laser group, while most of the tumor cells remained in other groups. At the same time, we sampled the tumor on the 14th day of tumor inoculation, and explored whether the specified treatment could induce apoptosis in the tumor area by Tunnel staining. The results of Fig. 7E show that AIE@HFn-scfv + Laser could effectively induce apoptosis of tumor cells (blue is DAPI signal, green is Tunnel signal), while AIE@HFn + Laser can induce apoptosis of some tumor cells, and other groups can not induce apoptosis of tumor cells. In addition, we evaluated the biosafety of AIE@HFn-scfv combined with laser in the treatment of early subcutaneous gastric cancer. We obtained the peripheral blood serum and main organs of mice in different treatment groups on the 6th day after laser irradiation, and judged the toxicity and side effects of AIE@HFn-scfv on tumor-bearing mice by detecting the related indexes of serum liver and kidney and the pathological changes of main organs. The blood biochemical data of Fig. 7F–M showed that there was no significant difference in the range of related biochemical parameters between AIE@HFn-scfv + Laser group and PBS group, and the HE staining results of different organs (Fig. 7N) showed that AIE@HFn-scfv + Laser group could not induce obvious pathological changes of heart, liver, spleen, lung and kidney. The above results proved that AIE@HFn-scfv + Laser could effectively heal early gastric cancer model mice without any side effects.

## 3 Discussion

The development of the AIE@HFn-scfv multifunctional imaging nanoprobe with dual targeting specificity for Claudin18.2 and CD71 represents a paradigm shift in gastric cancer theranostics. This innovative nanoplatform circumvents inherent limitations of conventional small-molecule theranostic agents through three complementary technological breakthroughs. First and foremost, its dual-targeting architecture capitalizes on the co-overexpression of CD71 and Claudin18.2 in gastric carcinoma cells, achieving a 25-fold enhancement in cellular internalization compared to single-target probes (Fig. 3F). This strategic design mitigates the challenge of heterogeneity-driven false negatives in early-stage tumors, as demonstrated by immunohistochemical analysis (Fig. 1B–D).

In comparison to existing clinically approved imaging agents such as FDG-PET/CT, which remain the cornerstone for staging and metabolic assessment in gastric cancer, AIE@HFn-scfv offers distinct advantages in terms of multimodal imaging capabilities and targeted theranostics. While FDG-PET provides whole-body metabolic information with high sensitivity, its specificity can be limited by inflammatory uptake and false positives. In contrast, AIE@HFn-scfv leverages dual-receptor targeting (CD71/Claudin18.2) to achieve a tumor-to-background ratio of approximately 2.5-fold higher than single-target probes (Fig. 4, Fig. 5F), enabling submillimeter tumor detection with high specificity. Furthermore, its NIR-II fluorescence and photoacoustic imaging capabilities allow for real-time, high-resolution visualization of superficial and deep-seated lesions with enhanced tissue penetration, complementing the depth penetration of PET while offering additional therapeutic potential via photothermal ablation. Although direct quantitative comparisons with clinical agents were beyond the scope of this preclinical study, the integrative design of AIE@HFn-scfv positions it as a promising complementary tool for sensitive detection and image-guided therapy, particularly in settings where receptor overexpression is confirmed.

Secondarily, the hemoglobin nanocage (HFn) scaffold integrates exceptional biocompatibility, prolonged systemic circulation exceeding 48 h (Fig. 4B), and high-capacity loading of AIEgens with >90 % encapsulation efficiency and pH-responsive release kinetics. The modular conjugation of 24 scfv antibodies per nanoparticle via Spycatcher/Spytag technology establishes a multivalent targeting platform unattainable with small-molecule agents, significantly enhancing binding avidity to tumor cells (Fig. 2A–D).

Thirdly, the multimodal theranostic capabilities of AIE@HFn-scfv seamlessly integrate NIR fluorescence imaging (R^2^ = 0.987 for tumor volume correlation, Fig. 6C), photoacoustic tomography (R^2^ = 0.935, Fig. 6D), and photothermal therapy (ΔT = 53.6 °C, Fig. 6E–F). This trifunctional design overcomes aggregation-caused quenching (ACQ) and the therapeutic limitations of single-modality probes. Collectively, these advancements enable submillimeter tumor detection (10 mm^3^ sensitivity), precise photothermal ablation with complete tumor elimination (Fig. 6A–B), and inherent biosafety profiles with no observed organ toxicity (Fig. 6F–N), positioning AIE@HFn-scfv as a next-generation solution for gastric cancer management.

A critical consideration in nanoprobe design is off-target accumulation, particularly given CD71 expression in hepatocytes [31]. The AIE@HFn-scfv nanoprobe addresses this challenge through a two-pronged strategy: leveraging HFn's intrinsic tumor-homing properties and implementing a dual-targeting mechanism. HFn's natural affinity for CD71, a receptor essential for iron-dependent tumor proliferation, ensures preferential tumor localization. The covalent conjugation of Claudin18.2-specific scfv antibodies via Spycatcher/Spytag technology introduces a second tumor-specific targeting axis, redirecting nanoparticles away from hepatic uptake and toward gastric cancer cells. This synergy results in a significantly improved tumor-to-liver signal ratio compared to non-functionalized controls, underscoring the advantage of dual-targeting over single-receptor approaches. Additionally, HFn's hollow nanostructure enables unparalleled AIEgens loading capacity, biocompatibility, and clinical scalability, surpassing synthetic nanoparticle platforms in translational potential.

Despite these advancements, several critical challenges warrant further investigation. Although the 808 nm laser used herein is suitable for subcutaneous and orthotopic models, its penetration depth is limited for deep-seated tumors. Future iterations incorporating NIR-II AIEgens (1000–1400 nm) could further enhance clinical translatability for deeply located malignancies. In addition, optimization of *in vivo* pharmacokinetics and biodistribution profiles remains essential to maximize therapeutic efficacy. Long-term safety assessments in preclinical models and eventual clinical trials will be necessary to validate its biosafety profile. Furthermore, continued innovation in imaging modality integration and therapeutic payload development will be required to fully realize the potential of this nanoplatform. In addition, while the probe shows high sensitivity for small-volume tumors, further studies in genuine early-stage or precancerous models are needed to fully validate its “early diagnostic” potential.

In conclusion, the AIE@HFn-scfv nanoprobe represents a significant milestone in gastric cancer theranostics by harmonizing dual-targeting specificity, multimodal imaging, and photothermal therapy. Its strategic design addresses key limitations of existing technologies while demonstrating superior performance in sensitive detection, precise ablation, and biosafety. With continued refinement and translational validation, this platform has the potential to redefine clinical approaches for this devastating disease, paving the way for next-generation precision oncology.

## 4 Experimental Section

### 4.1 Bioinformatics analysis

The expression data of Claudin18 and TFRC in different cancer types and corresponding paracancerous tissues were obtained from the online analysis tool of Timer 2.0 database (http://timer.cistrome.org/). Claudin18 and TFRC expression data in gastric cancer patients with different stages were obtained from the online analysis tool of the UALCAN database (https://ualcan.path.uab.edu/index.html). Immunohistochemical data of Claudin18 and TFRC in gastric cancer patients were obtained from Human protein atlas database (https://www.proteinatlas.org/).

● **Pre-processing of scRNA-seq data**: The scRNA-seq data for the gastric cancer samples (accession number GSE167297) were obtained from the GEO database (http://www.ncbi.nlm.nih.gov/geo), retaining only the deep layer of gastric cancer tissues from the original study. The raw output data were processed with the “Seurat” R package (version 4.3.0; http://satijalab.org/seurat/) for each individual sample. In each sample, low-quality cells that did not meet the following criteria were excluded: (1) 1000 ≤ nCounts ≤100000; (2) 200 ≤ nFeatures ≤7500; (3) percentage of mitochondrial genes ≤20; and (4) percentage of ribosomal genes ≤50. Also, to avoid the effect of doubled cells, we excluded from the data doubled cells identified using “scDblFinder” (version 1.12.0; https://plger.github.io/scDblFinder/) R package.
● **Data integration and the dimensionality reduction**: All samples were merged with the “AddSamples” function into one Seurat object. The merged Seurat object was normalized and scaled by regressing out UMI count and percentage of mitochondrial genes. For dimensionality reduction, the top 3000 highly variable genes (HVGs) were determined using the “FindVariableGenes” function. Dimensionality reduction was then performed using PCA and UMAP plots were generated by the “RunUMAP” function with the first 100 PCs as input, determined by visualizing the drop off in PC variance explained using the “ElbowPlot” function in Seurat. The batch effects were removed by the “Harmony” R package (version 1.2.0; https://github.com/immunogenomics/harmony) based on the top 50 PCA components identified.
● **Cell-clustering and annotation**: The clustering analysis was performed based on the integrated joint embedding produced by Harmony with the Louvain algorithm after computing a shared nearest-neighbor graph with the Louvain algorithm that was implanted in the “FindClusters” function of the Seurat package. The identified clusters were visualized on the 2D map produced with the UMAP method. To annotate the cell clusters, DEGs with high discrimination abilities between the groups were identified with the “FindAllMarkers” function in Seurat using the default non-parametric Wilcoxon rank sum test with Bonferroni correction. The cell groups were annotated based on the DEGs and the well-known cellular markers from the literature. We then annotated clusters based on the average expression of genes in the following major cell types: epithelial cells (EPCAM and KRT18), myeloid cells (CD14, CD68, and CD163), T cells (PTPRC and CD3D/E/G), B cells (CD79A, CD19, and MS4A1), and fabroblasts (COL1A2, PDGFRA, and DCN), mast (CPA3 and TPSAB1), endothelial cells (ENG and VWF), plasma cells (CD79A and SDC1) and cycling cells (TOP2A and MKI67).

### 4.2 Preparation of HFn-Sc, St-scfv, HFn-scfv

HFn-Sc consisted of 100 % heavy-chain subunits, St-scfv was firstly purifed by metal ion affinity chromatography after obtained from *Escherichia coli*, then these protein were purified using a fast protein liquid chromatography system with a HiLoad 16/60 Superdex 200 pg column (General Electric Healthcare, NY, USA) and concentrated using a concentrator tube with a 10 kDa molecular weight cutoff (Millipore, USA). The protein content of the nanoparticles was quantified using an mp06667-CBQCA protein quantitation kit (Invitrogen Corporation, USA). The HFn-scfv was purified with fast protein liquid chromatography system after after incubation of HFn-Sc and St-scfv at equimolar concentrations, and stored at 4 °C overnight.

### 4.3 Preparation of AIE@HFn, AIE@HFn-scfv

The AIEgens BBTDT-BT-TPA (BBT) was purchased from Xianfeng Nanotechnology Co., Ltd (Cat # XFAI06). The AIEgen BBTDT-BT-TPA emits in the NIR-I region (∼935 nm). Due to the filter configuration limitations of our confocal microscope for this wavelength, we co-encapsulated the DiD dye (emission ∼665 nm, purchased from MedChemExpress (Cat # HY-D1028)) with the AIEgen to enable direct visualization of cellular uptake and localization via confocal microscopy. According to the molar concentration ratio of HFn-Sc to AIE and DiD of 1:20:10, the HFn-Sc, AIE (dissolved in DMSO) and DiD (dissolved in DMSO) was dissolved in PBS solution at pH = 2.0, and then the pH was adjusted to 7.0. *To preserve structural integrity, ferritin disassembly and reassembly were performed using a stepwise pH adjustment protocol (pH 2.0 to pH 7.0) followed by gentle crosslinking, as previously described* [32]*.* Then, AIE@HFn was obtained by purification using a fast protein liquid chromatography system. If AIE@HFn-scfv was required, an equimolar concentration of AIE@HFn and St-scfv should be incubated in PBS solution overnight at 4 °C, and all of the nanoparticles were purified using a fast protein liquid chromatography system with a HiLoad 16/60 Superdex 200 pg column (General Electric Healthcare, NY, USA) to obtain AIE@HFn-scfv, and then concentrated to the desired concentrations.

### 4.4 Material characterization

Based on the principle of DLS, a Malvern particle size analyzer (Zetasizer Nano-ZS90 (Malvern Instruments, Worcestershire, UK)) was used to detect the hydrated particle size and zeta potential of nanoparticles, and TEM (HT7700-type TEM (Hitachi High Technology Company)) was used to analyze the uniformity and particle size of HFn, HFn-scfv and AIE@HFn-scfv. TEM images were uploaded to the Quick word recognition application, and the recognition quantity module was used for statistical analysis of dark spots in the ROI region of the uploaded images. The ultraviolet–visible spectra were recorded on an Agilent Cary 60 spectrophotometer (Agilent Technologies, Santa Clara, USA). The NIR-I fluorescence emission spectra were performed on an Edinburgh FLS980 spectrophotometer with 808 nm laser diode. The liquid temperature were acquired using an Optris PI infrared camera (Optris Infrared Sensing LLC., Portsmouth, NH, USA). For PA imaging, the incubation solutions at each time point were loaded into a fine bore polythene tube (0.86 mm OD, 1.27 mm OD). The tubes were then sealed and immersed in water. The PA images at both 700 and 808 nm were acquired on the Vevo 2100 LAZR system (FUJIFILM VisualSonics).

### 4.5 Stability evaluation

AIE@HFn-scfv were incubated with PBS, 10 % FBS, 10 % plasma, and 1 % Triton for 3 h at 37 °C. The stability of AIE@HFn-scfv was analyzed and evaluated using SDS-polyacrylamide gel electrophoresis (SDS-PAGE) with an 8 % semi-native gel and a custom-made optical fluorescence imaging system that had been fitted with a green filter set (excitation filter: 469/35 nm; emission filter: 510/42 nm), a near-infrared filter set (excitation filter: 640/40 nm; emission filter: 700/40 nm), and a 150 W xenon lamp (Crownteck, Inc., PA, USA).

### 4.6 Cell lines

Hela cells, MC-38 cells, L02, MGC803 and HEK293 cells were purchased from the China Center for Type Culture Collection (Wuhan, China) and cultured in complete medium containing 10 % (*V*/*V*) FBS and 1 % (*V*/*V*) penicillin/streptomycin. HEK293 expressing low Claudin18.2 and HEK293 expressing high Claudin18.2 was obtained by transduced with lentiviral vectors coding for Claudin18.2. All cells were maintained in an incubator at 37 °C in a humidified atmosphere of 95 % and 5 % CO_2_. The cell lines were examined for mycoplasma and viruses according to the FELASA guidelines by Charles River Research Animal Diagnostic Services (CR RADS.Wilmington, MA, USA: Mouse essential panel) before *in vivo* transplantation.

### 4.7 Western blotting

The cell culture medium was treated with RIPA lysis buffer, and protein separation was carried out on an 8 % SDS-PAGE gel. Subsequently, membrane transfer was performed using a 0.2 μm PVDF membrane (Millipore), followed by antibody incubation. The Claudin18.2 antibody (Catalog number: 66167-1-Ig), CD71 antibody (Catalog number: 66180-1-Ig), and GAPDH antibody (Catalog number: 60004-1-Ig) were all purchased from Proteintech. All images were acquired using the ChemiDoc Imaging System (Bio-Rad).

### 4.8 Detection of cellular uptake by using confocal microscopy

HFn and HFn-scfv were labeled by 5/6-Fluorescein isothiocyanate in sodium bicarbonate solution (pH = 9.0), and equimolar concentrations of AIE@HFn-FITC and AIE@HFn-scfv-FITC were co-incubated with MGC803 inoculated in confocal petri dishes in complete medium for 3 h, then washed twice by using PBS before staining the nuclei of the cells with Hochest dye, and the fluorescence intensities of FITC and DAPI were obtained using a turntable confocal microscope (PerkinElmer) and all images were processed by using the image J software.

### 4.9 Detection of cellular uptake by flow cytometry

HFn-Sc, St-scfv and HFn-scfv were labeled by 5/6-Fluorescein isothiocyanate in sodium bicarbonate solution (pH = 9.0). These proteins were infused with different cells in complete medium for 3 h, then washed twice by using PBS. The FITC intensity of different cells were obtained by using flow cytometry (cyto flex).

### 4.10 Cytotoxicity assay

The cell cytotoxicities of different treatment were analyzed using the CCK-8 Kit following the kit instructions. First, 5 × 10^3^ MGC803 cells per well were placed in the 96-well plates in RPMI medium (200 μL). After incubation for 24 h, the cells culture medium was replaced with fresh RPMI medium containing different concentrations (0, 10, 20, 30, 60, 90, 120, 160 μg/mL) of HFn, HFn-scfv, AIE@HFn, or AIE@HFn-scfv for further incubation of 24 h. Then, the cells were washed with PBS buffer, and the RPMI medium (100 μL) containing CCK-8 (10 μL) was added to each well of 96-well plates and incubated for 2 h. After incubation, the orange formazan product from CCK-8 was produced due to the reduction of WST-8 by dehydrogenases in live cells. After that, the cell cytotoxicity was evaluated by measuring the absorbance per well at 450 nm.

### 4.11 Cell viability assay

Inoculate 1 × 10^5^ MGC803 cells into a 12-well cell culture plate and incubate for 24 h. Then incubate with the indicated treatment groups for another 24 h. Discard the supernatant and wash twice with PBS, followed by fixation with 4 % paraformaldehyde for 30 min. Then add 0.1 % crystal violet staining for 30 min. Finally, the supernatant was discarded, washed 1 time with PBS, dried and photographed.

### 4.12 Animals

Balb/C nude mice were purchased from Hunan SJA Laboratory Animal Co. Ltd. (Hunan, China). All animal studies were conducted in compliance with protocols approved by the Hubei Provincial Animal Care and Use Committee and followed the experimental guidelines of the Animal Experimentation Ethics Committee of Huazhong Agricultural University, and the animal experimentation ethics number was HZAUMO-2024-0175. In the housing facility, humidity and temperature were controlled, and a 12 h light-dark cycle was set. Water and food were freely available. Animals were housed in the animal facility for at least 1 week before subcutaneous injection of indicated treatment. Male mice that were 6–8 weeks old with a weight of 18–25 g were included in the *in vivo* experiments.

### 4.13 Establishment of orthotopic GC mouse models

Randomly selected balb/c-nu mice (6−8 weeks, male) utilized for MGC803-RFP cells (4 × 10^6^ cells per mice) implantation to establish an orthotopic GC mice model. MGC803-lucferase (Luc) cells cells were cultured and mixed 1:1 with Matrigel and PBS, temporarily stored on ice. Anesthetized mice by pentasorbital sodium, opened abdominal cavity and injected MGC803-luc cells under the gastric plasma membrane layer, followed by closing the abdominal cavity the returning the mice to the SPF environment for further rearing. Bioluminescence imaging was performed on the mice after surgery for indicating tumor implantation. Body weights of all the mice were recorded during experiments. Three successfully constructed mice per group were used for subsequent experiments (see ).

### 4.14 In vivo and in vitro fluorescence imaging and PA imaging of tumors

The tumor-bearing mice were randomly divided into different groups (n = 5) and injected intravenously through the tail vein with the indicated treatment which was dissolved in PBS (0.2 mg total protein for each mouse). IVIS Spectrum imaging system was applied to detect the fluorescence at different time points post-injection of indicated treatment. The major organs (including heart, liver, spleen, lung, kidney) and tumors were harvested for imaging analysis and fluorescence intensity was measured using the IVIS system. Similarly, the photoacoustic imaging of tumor-bearing mice was conducted with inVision128 multispectral optoacoustic tomographic (MSOT) imaging system (iThera Medical GmbH) at various time points after injection of indicated treatment. The PA imaging of the tumor was analyzed using the Vevo LAB software.

### 4.15 Tissue multicolor immunofluorescent staining

Tumor tissues and liver tissues were fixed, embedded in paraffin, and sectioned with a microtome. The sections were then dewaxed and hydrated routinely. Antigen retrieval was achieved by applying a Tris-EDTA Buffer solution, and endogenous peroxidases were quenched using 3 % H_2_O_2_. Samples were subsequently blocked with normal goat serum. Subsequently, DAPI was applied for 20 min at room temperature. Finally, tissue immunofluorescence was analyzed using the PE Vectra (PerkinElmer).

### 4.16 Assessment of the photothermal therapeutic effect of AIE@HFn-scfv

For *in vivo* PTT, The tumor-bearing mice were randomly divided into different groups (n = 8) and injected intravenously through the tail vein with the indicated treatment which was dissolved in PBS (0.2 mg total protein for each mouse). tumor-bearing mice were irradiated with an 808-nm laser (0.8 W/cm2) for 10 min. Tumor temperature and thermographic images were acquired using an Optris PI infrared camera (Optris Infrared Sensing LLC., Portsmouth, NH, USA). The tumor volumes after treatments were measured using a caliper, and the volume was calculated according to the formula (*a* × *b*^2^)/2, where *a* and *b* are the long and short diameters of the tumor, respectively. The relative tumor volume (*V*/*V*0, where *V*0 denotes the initial tumor volume, i.e., day 0) was used to monitor tumor growth. The tumor volume and body weight were recorded every 2 days and 3 days during the therapy period.

### 4.17 Histopathological assays

Tumor tissues or other organs from mice were collected, immediately fixed in 10 % (*m/V*) neutral-buffered formalin without inflation and embedded in paraffin. Approximately 5-μm sections were cut and mounted on slides. Histopathological changes were examined using H&E staining and visualized under a light microscope. H&E-stained tumor tissue sections were blindly examined and scored by trained histopathologists.

### 4.18 TUNEL staining

Cell apoptotic was also verified by detecting the DNA fragmentation of cells after indicated treatment on the TUNEL assay. Tumor tissues were fixed, embedded in paraffin, and sectioned with a microtome. The sections were then dewaxed and hydrated routinely. Antigen retrieval was achieved by applying a Tris-EDTA Buffer solution, and endogenous peroxidases were quenched using 3 % H_2_O_2_. Samples were subsequently blocked with normal goat serum. Finally, the cells were stained by the TUNEL Kit and immediately observed and imaged by CLSM.

### 4.19 Biosafety assessment

One month after two injections of vaccine in the tail root, peripheral blood was obtained through the orbital venous plexus, and mouse serum was obtained by 5000 g centrifugation. Liver and kidney toxicity was analyzed using a blood biochemical detector, and H&E staining was performed to evaluate the degree of organ damage and immune cell infiltration.

### 4.20 Statistical analysis

The unpaired two-tailed Student's t-test to compare the differences between the two groups was used, while survival rates were evaluated with the log-rank Mantel-Cox test using GraphPad Prism 7 software. Repeated measurements of tumor volume growth were compared using a One-way analysis of variance (ANOVA). Flow cytometry data were analyzed using FlowJo.10. Significant differences between the groups are indicated by ∗p < 0.05, ∗∗p < 0.01, and ∗∗∗p < 0.001, and NS, not significant.

## CRediT authorship contribution statement

**Junjian Deng:** Writing – review & editing, Writing – original draft, Methodology, Investigation, Formal analysis, Data curation. **Zengxing Zhang:** Visualization, Validation, Software, Resources, Formal analysis, Data curation. **Kejun Li:** Visualization, Supervision, Resources, Project administration. **Yongbin Zheng:** Writing – review & editing, Writing – original draft, Visualization, Methodology. **Yongfa Zheng:** Writing – review & editing, Writing – original draft, Methodology, Investigation, Funding acquisition.

## Ethics approval

All animal studies were conducted in compliance with protocols approved by the Hubei Provincial Animal Care and Use Committee and followed the experimental guidelines of the Animal Experimentation Ethics Committee of Huazhong Agricultural University, and the animal experimentation ethics number was HZAUMO-2024-0175.

## Consent for publication

All the authors reviewed and accepted the contents of the article.

## Funding declaration

This study was supported by grants from the Research project of 10.13039/100017958Hubei Provincial Health Commission (No. WJ2023Q010) and the Natural Science Foundation General Project of Hubei Province (No.2023AFB724).

## Declaration of competing interest

The authors declare that they have no known competing financial interests or personal relationships that could have appeared to influence the work reported in this paper.

## Data Availability

Sample sizes were predetermined based on previous experience using at minimum three groups of mice, and all experiments were replicated at least twice to confirm findings. No animals or potential outliers were excluded from the data sets presented in this study. The data used and analyzed during the current study are available from the corresponding author on reasonable request.
