# Supplementary material for: Ferritin-nanocaged aggregation-induced emission nanoaggregates for integrated sensitive detection and treatment of gastric cancer
Source: Mater Today Bio. 2026 Jan 6;36:102769. doi: 10.1016/j.mtbio.2026.102769 (PMC12818144; doi:10.1016/j.mtbio.2026.102769)
Supplement: Multimedia component 1 [file mmc1.docx]

**Supporting information**

**Ferritin-Nanocaged Aggregation-Induced Emission Nanoaggregates for integrated early diagnosis and treatment of gastric cancer**

Junjian Deng^1,#^, Zengxing Zhang^1,#^, Kejun Li^1^, Yongbin Zheng^2^**🖂**, Yongfa Zheng^1^**🖂**

^1^Cancer Center, Renmin Hospital of Wuhan University, Wuhan 430060, China.

^2^Department of Gastrointestinal Surgery, Renmin Hospital of Wuhan University, Wuhan 430060, China.

#These authors contributed equally: Junjian Deng, Zengxing Zhang.

**🖂**e-mail:zyf0322@126.com (Y. Zheng), yongbinzheng@whu.edu.cn (Y. Zheng)

Table1. the protein sequence used in the study.

| Protein name | Peptide sequence |
| --- | --- |
| Spycatcher003-  Ferritin | VTTLSGLSGEQGPSGDMTTEEDSATHIKFSKRDEDGRELAGATMELRDSSGKTISTWISDGHVKDFYLYPGKYTFVETAAPDGYEVATPIEFTVNEDGQVTVDGEATEGDAHTGGGGSGGGGSMTTASTSQVRQNYHQDSEAAINRQINLELYASYVYLSMSYYFDRDDVALKNFAKYFLHQSHEEREHAEKLMKLQNQRGGRIFLQDIKKPDCDDWESGLNAMECALHLEKNVNQSLLELHKLATDKNDPHLCDFIETHYLNEQVKAIKELGDHVTNLRKMGAPESGLAEYLFDKHTLGDSDNESHHHHHH. |
| Spytag003-scfv | RGVPHIVMVDAYKRYKGGGGSGGGGSDIVMTQSPSFLTVTAGEKVTLNCKSSQSLLNSGNQKSYLTWYQQKPGQPPKLLIYWASTRESGVPDRFIGSGSGTDFTLTISSVQAEDLAVYYCQNNYFYPVTFGAGTKLELKGGGGSGGGGSGGGGSEVQLQQSGPELVKPGASVKISCQASGYTFTDYYMNWVKQSHVKSLEWIGHINPKNGGTNYNQNFKDKATLTVDKSSSTAYMELRSLTSEDSAVYYCARIYYGNSFVYWGQGTLVTVSAHHHHHH |


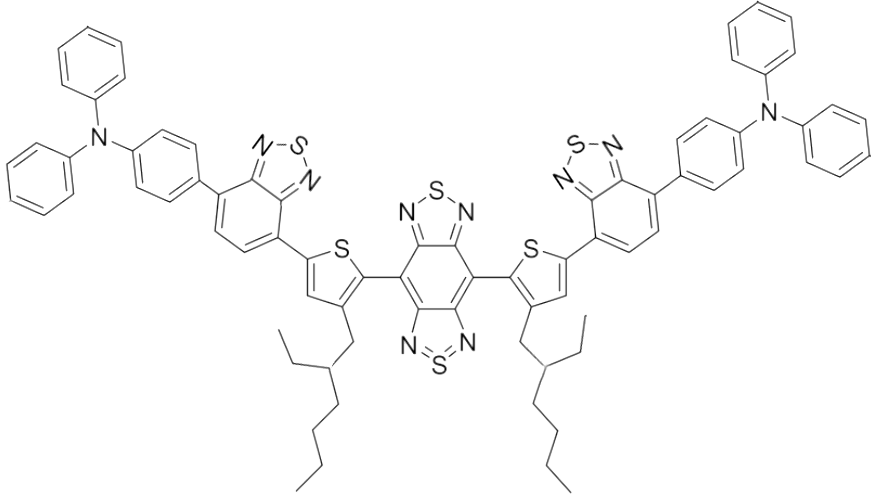


Figure S1. Structural diagram of BBTDT-BT-TPA molecule.


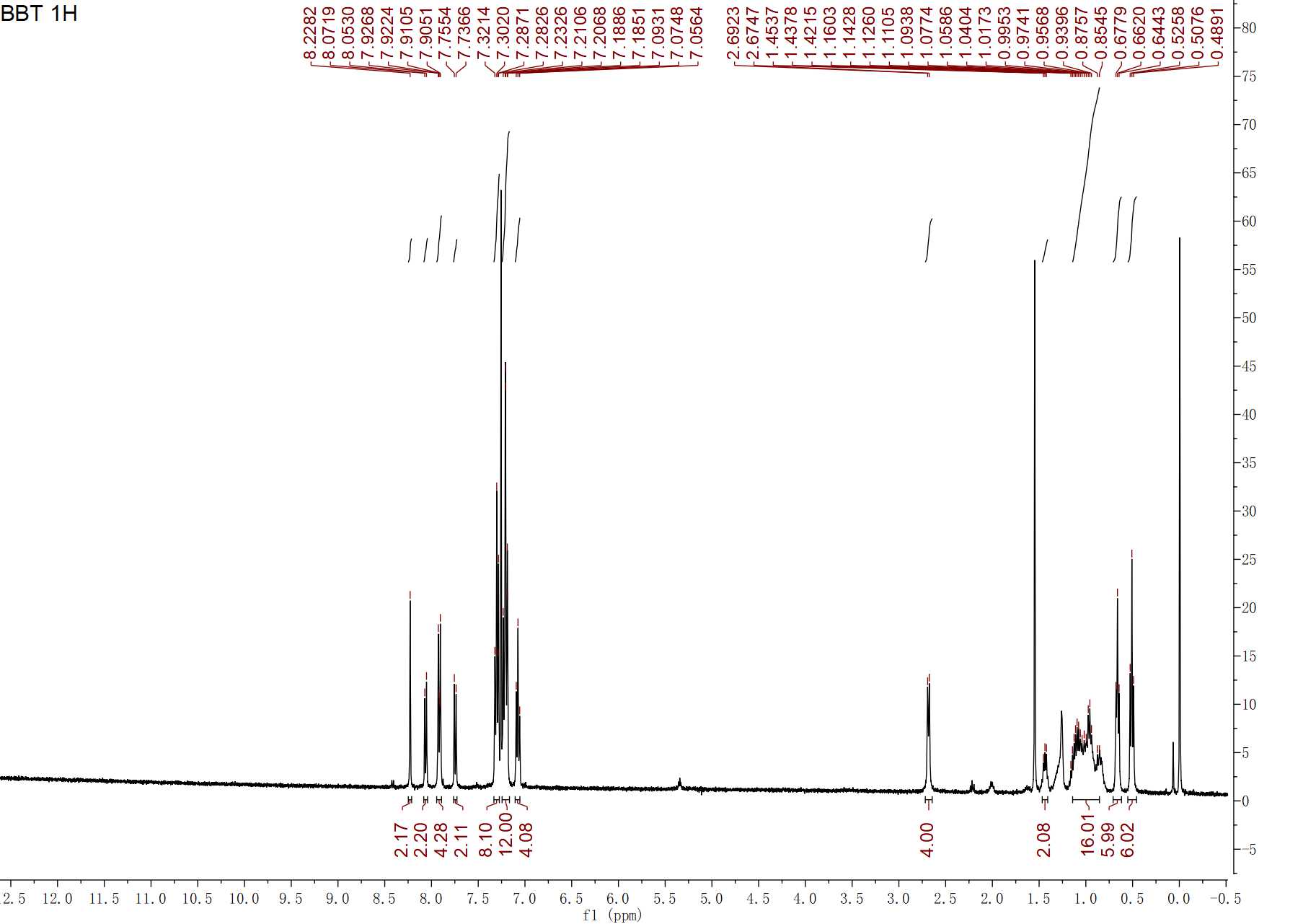


Figure S2. ^1^H NMR of BBTDT-BT-TPA in CDCl_3_. ^1^H NMR (400 MHz, CDCl_3_-d) δ 8.23 (s, 2H), 8.06 (d, J=7.4 Hz, 2H), 7.92 (d, J=8.7 Hz, 4H), 7.7 (d, J = 8.6 Hz, 2H), 7.30 (t, J=8.0 Hz, 8H), 7.23-7.19 (m, 12H), 7.07 (t, J=7.3 Hz, 4H), 2.68 (d, J=7.0 Hz, 4H), 1.45-1.42 (m, 2H), 1.16-0.85 (m, 16H), 0.66 (t, J = 6.7 Hz, 6H), 0.51 (t, J = 7.3 Hz, 6H).


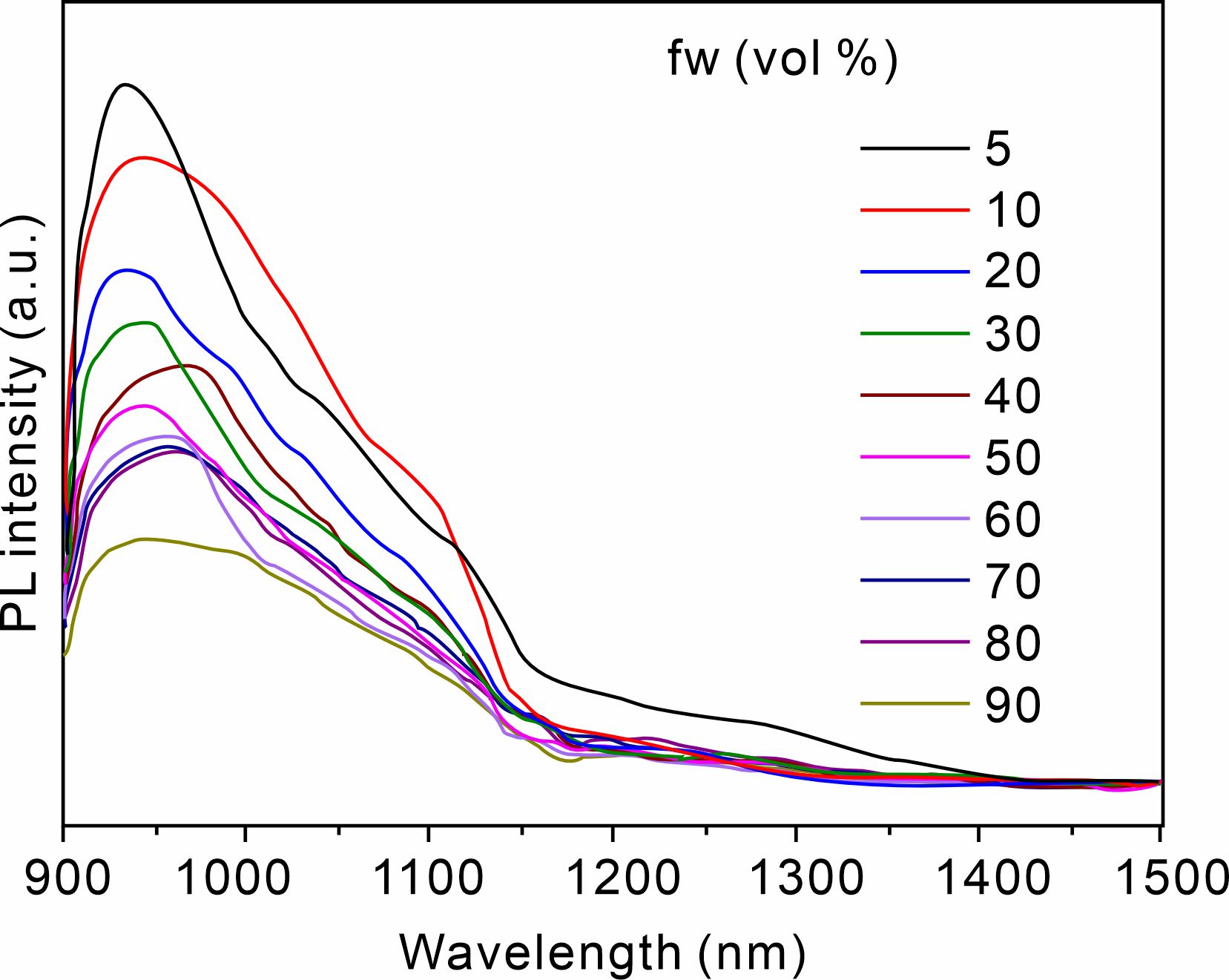


Figure S3**.** PL spectra of BBTDT-BT-TPA (10 μg/mL) in water−acetone mixtures with different acetone fractions (f_w_).


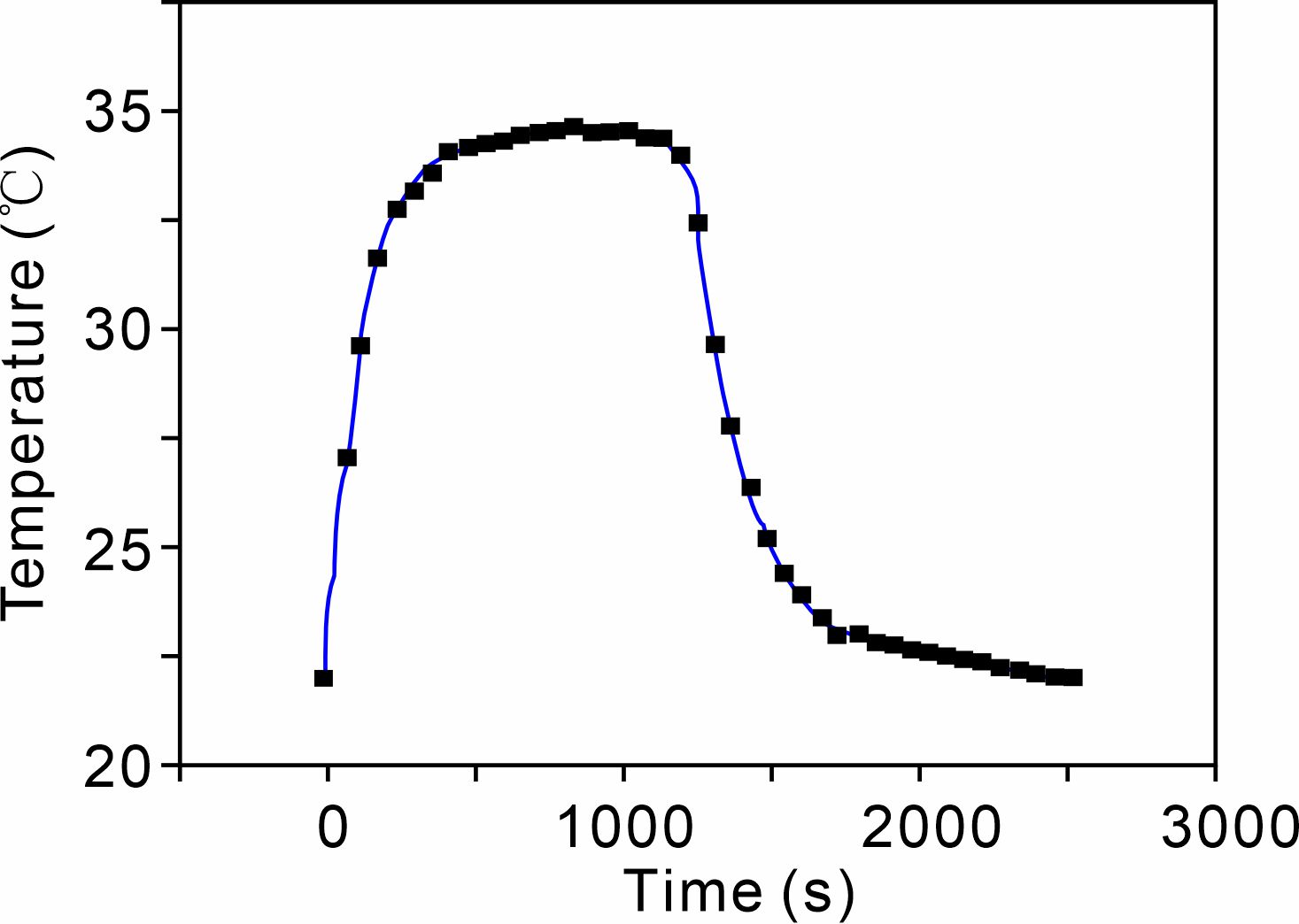


Figure S4. Monitoring the photothermal effect of liquid blend of BBTDT-BT-TPA under exposure to the NIR laser.


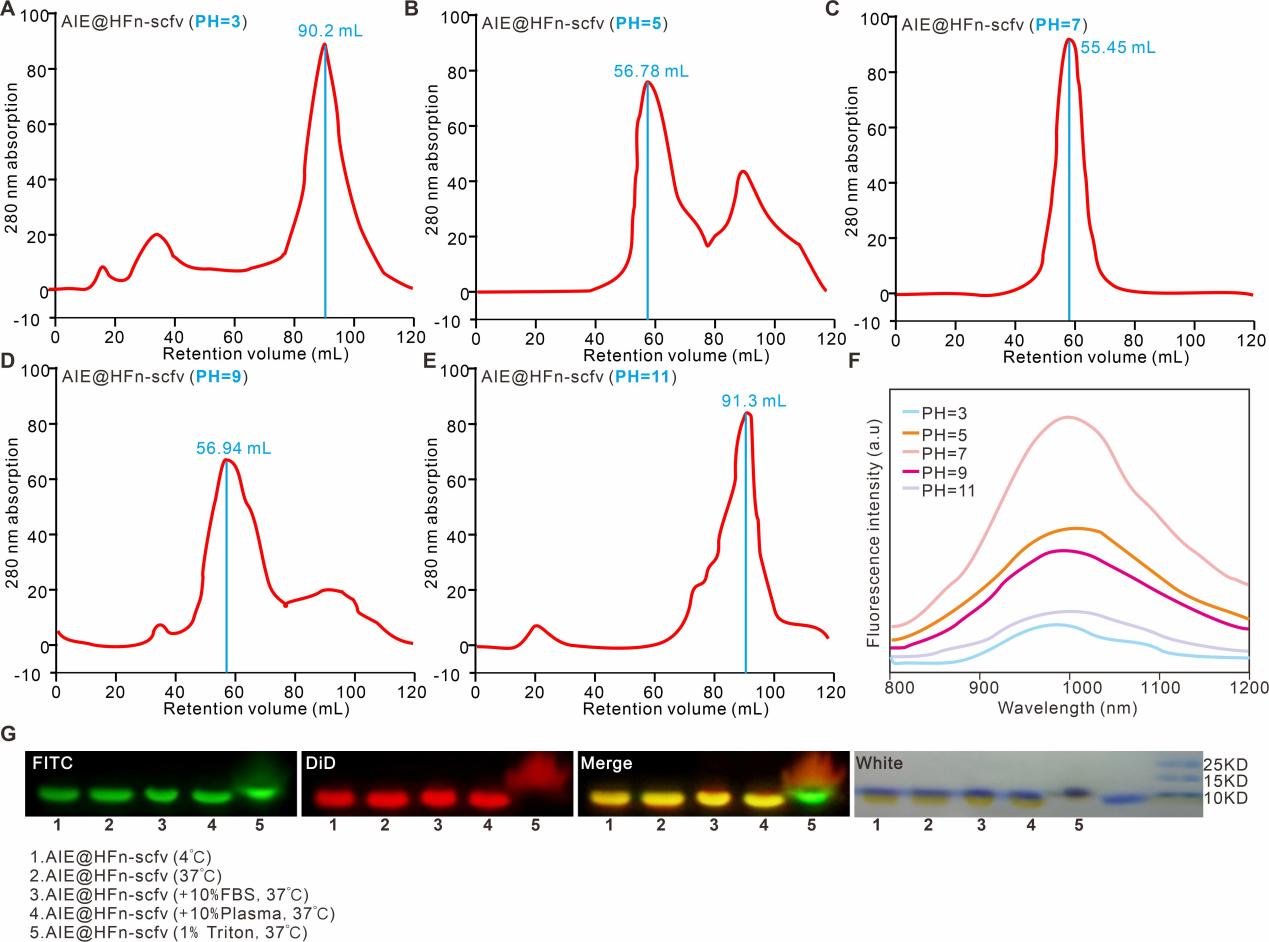


Figure S5. Characterization of the stability of AIE@HFn-scfv in aqueous solutions with different pH or serum-containing. (A–E) Size-exclusion chromatography profiles of AIE@HFn-scfv at pH 3, 5, 7, 9, and 11. (F) Fluorescence intensity changes of AIE@HFn-scfv under different pH conditions. (G) Stability analysis of AIE@HFn-scfv via under various conditions (4 °C, 37 °C, +10% FBS, +10% plasma, +1% Triton) for 24h. HFn-FITC (green), DiD (red), and merged fluorescence signals.


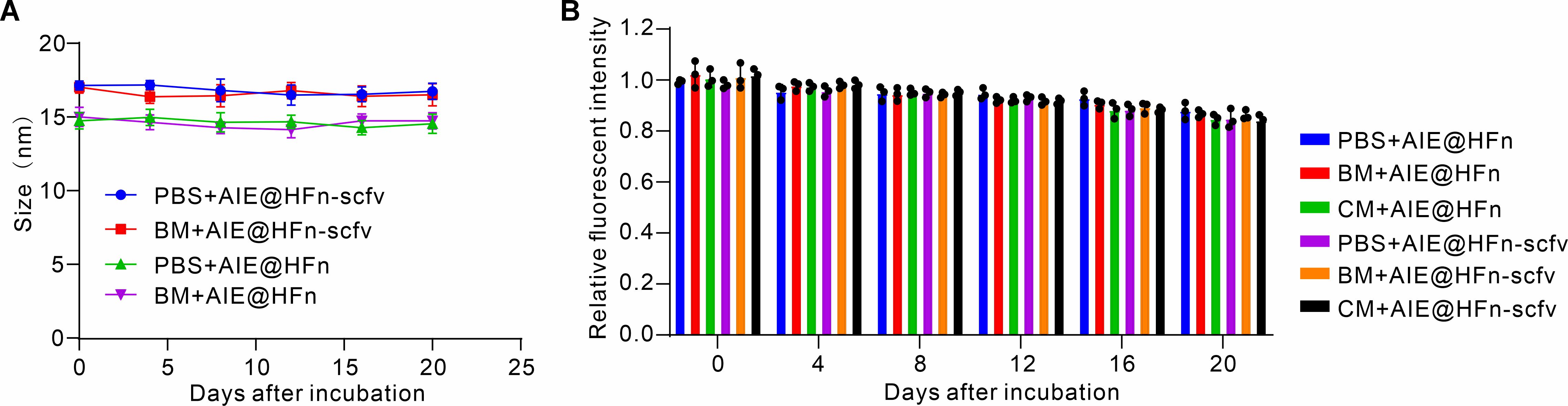


Figure S6. The size distribution and fluorescence activity of AIE@HFn and AIE@HFn-scfv after incubated with indicated treatment for 20 days.


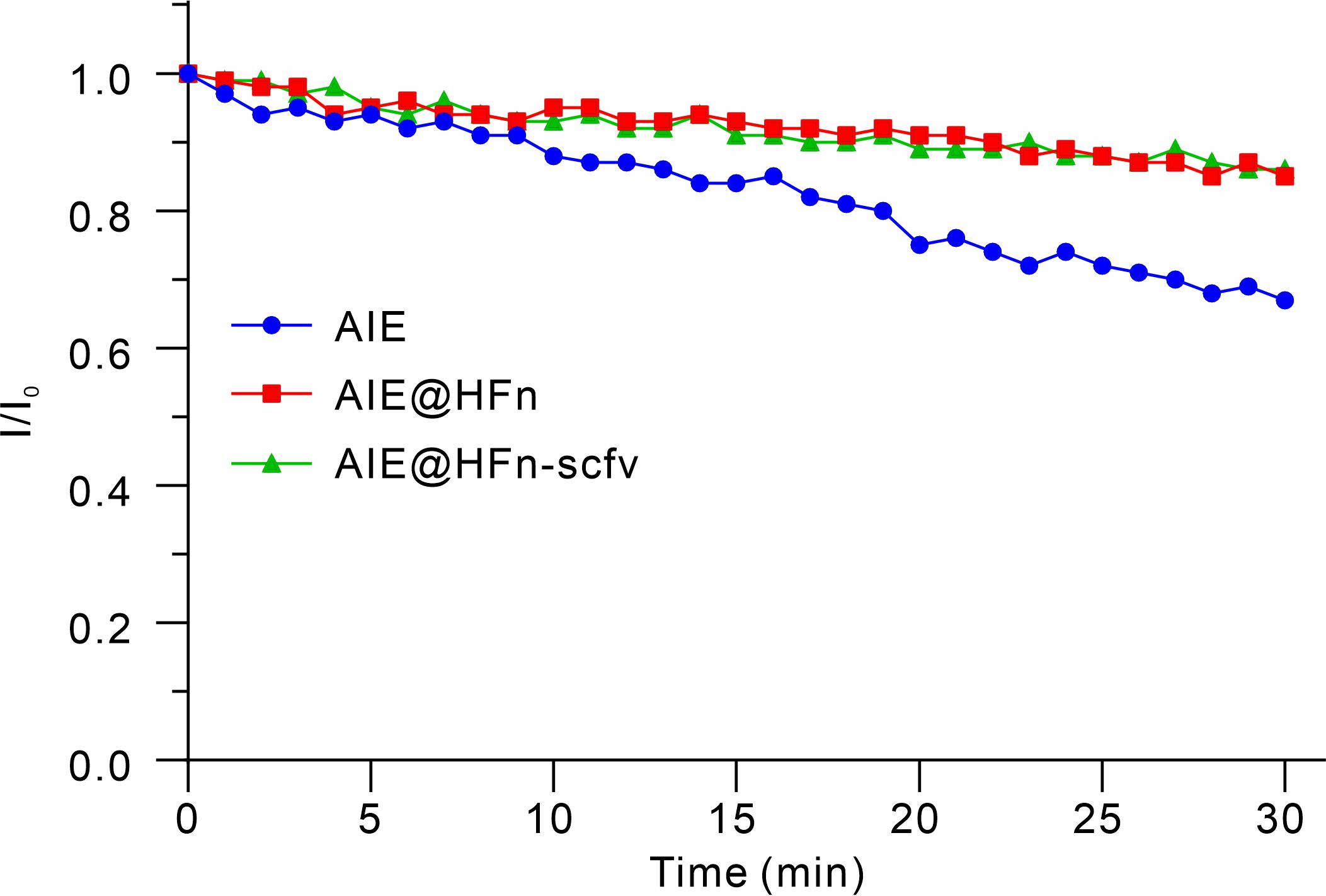


Figure S7**.** Plots of relative photoluminescence intensity (*I*/*I*_0_) of AIEgens, AIE@HFn, and AIE@HFn-scfv at corresponding emission peaks under continuous 808 nm laser irradiation (0.8 W/cm^2^), where *I*_0_ and *I* are the fluorescence peak intensity before and after laser irradiation, respectively.


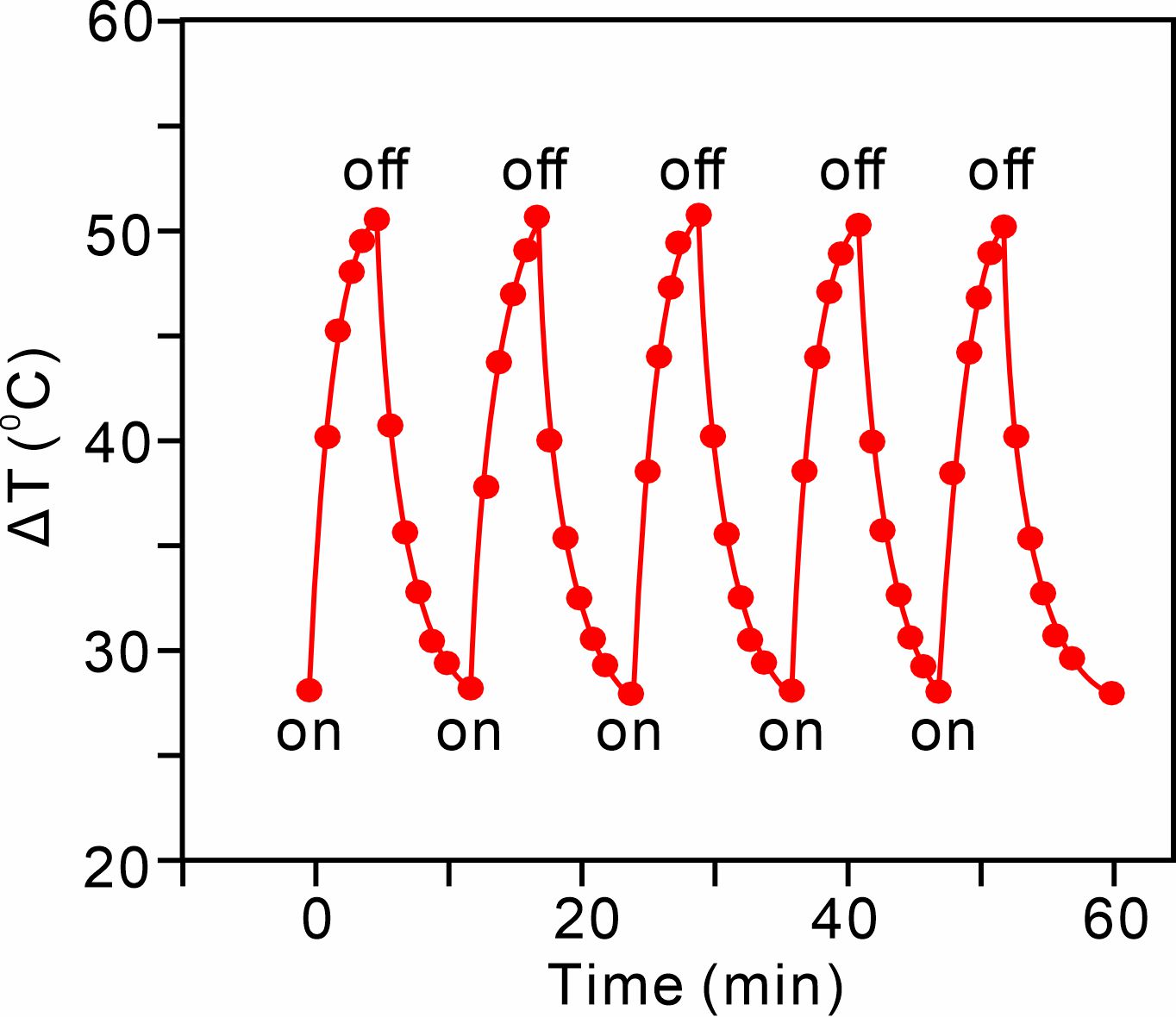


Figure S8. Photothermal performance of the AIE@HFn-scfv solutions for five laser on/off cycles.


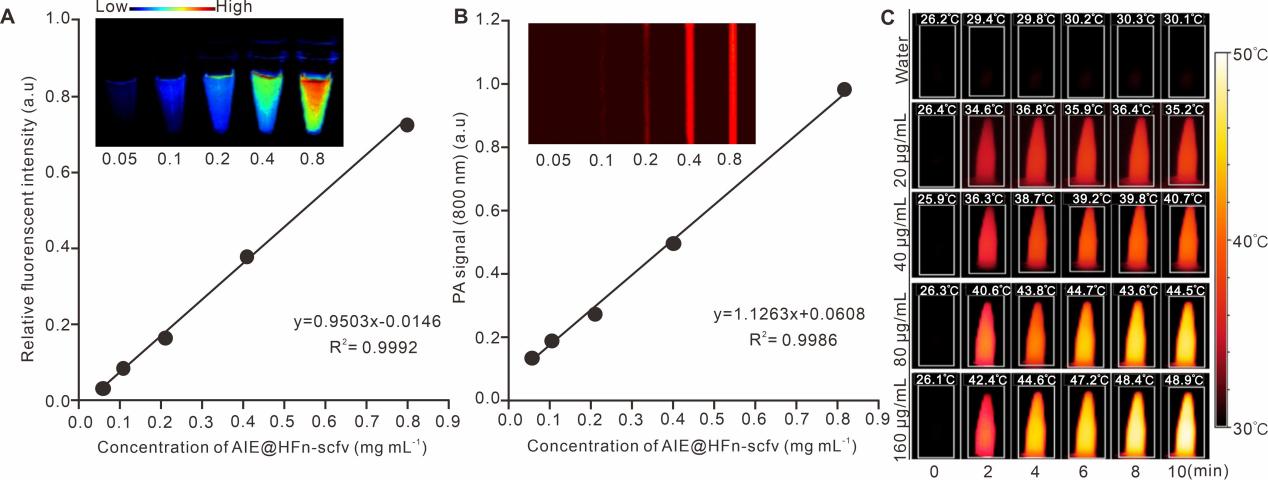


Figure S9. Evaluate the *in vitro* fluorescence, photoacoustic imaging and photothermal effect of AIE@HFn-scfv. (A) Concentration-dependent relative fluorescence intensity of AIE@HFn-scfv. (B) Linear relationship between AIE@HFn-scfv concentration and PA signal at 800 nm. (C) Photothermal effect images of AIE@HFn-scfv at different concentrations under laser irradiation.


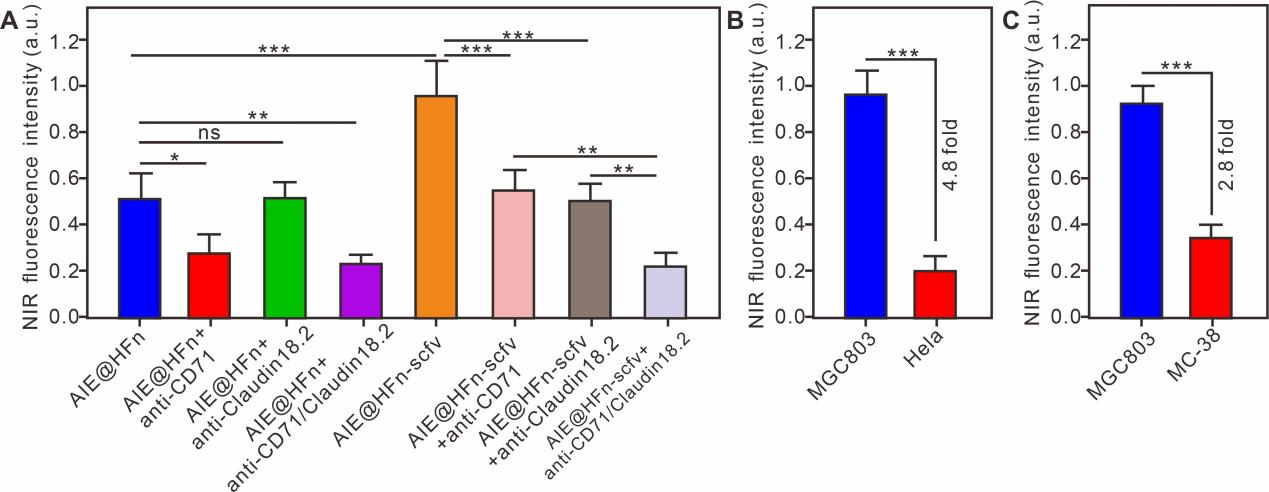


Figure S10. Statistics of the NIR florescence intensity in different group. (A) NIR fluorescence intensity comparison of AIE@HFn, AIE@HFn-scfv, and other groups. (B–C) Targeting specificity in MGC803 vs. Hela (B) and MC-38 cells (C). The fluorescence intensity of all groups has been normalized, and statistical analysis was performed using one-way ANOVA with Tukey's multiple comparison test for (A) or unpaired T test for (B) and (C). Data are presented as the mean ± SEM. **P* < 0.05,***P* < 0.01, ****P* < 0.001, and NS: not significant.


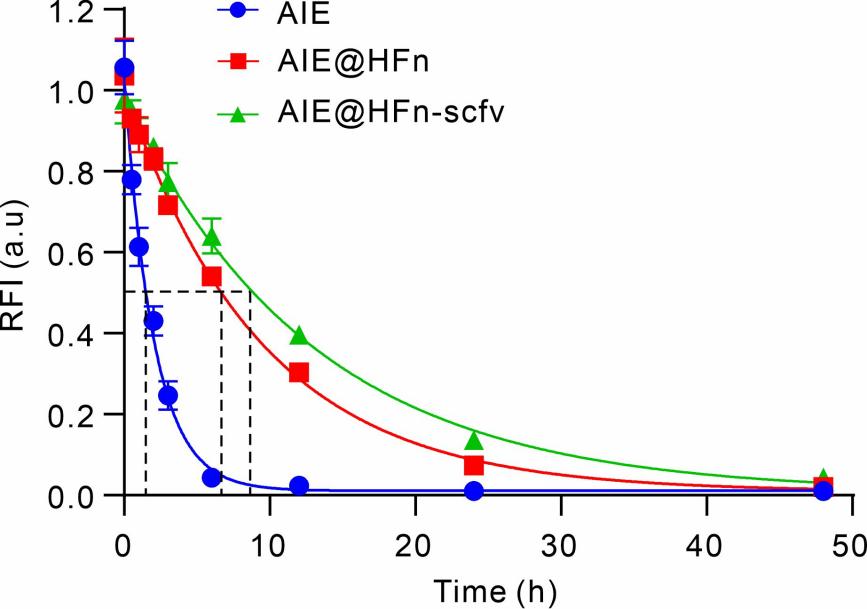


Figure S11. Evaluate the blood half-life of AIE, AIE@HFn, and AIE@HFn-scfv. After intravenous injection of AIE, AIE@HFn, and AIE@HFn-scfv at equal fluorescence intensity, peripheral blood samples from different groups of mice were obtained at different time points through orbital venous plexus blood collection and other methods. The blood half-life of AIE, AIE@HFn, and AIE@HFn-scfv was calculated based on the single-phase exponential decay of fluorescence.


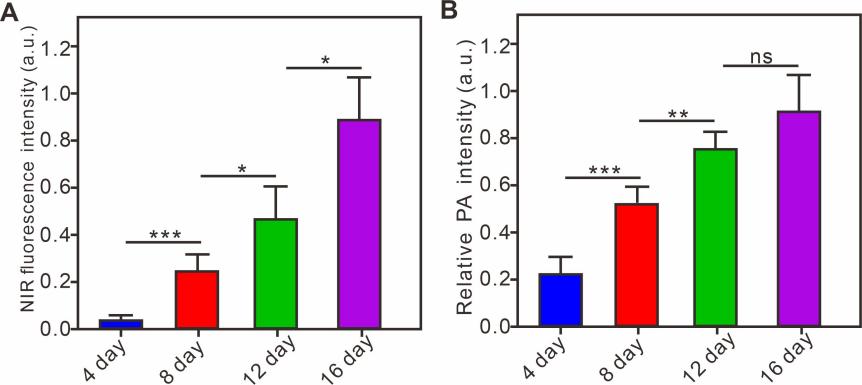


Figure S12. *In vivo* statistics on the contrast effect of AIE@HFn-scfv on fluorescence imaging and PA imaging of tumors with different days of growth. (A) Time-dependent NIR fluorescence intensity and (B) relative PA intensity of AIE@HFn-scfv from day 4 to 16. The fluorescence intensity and PA intensity of all groups has been normalized, and statistical analysis was performed using one-way ANOVA with Tukey's multiple comparison test. Data are presented as the mean ± SEM. **P* < 0.05,***P* < 0.01, ****P* < 0.001, and NS: not significant.
